# Histone Methyltransferase MMSET/NSD2 Alters EZH2 Binding and Reprograms the Myeloma Epigenome through Global and Focal Changes in H3K36 and H3K27 Methylation

**DOI:** 10.1371/journal.pgen.1004566

**Published:** 2014-09-04

**Authors:** Relja Popovic, Eva Martinez-Garcia, Eugenia G. Giannopoulou, Quanwei Zhang, Qingyang Zhang, Teresa Ezponda, Mrinal Y. Shah, Yupeng Zheng, Christine M. Will, Eliza C. Small, Youjia Hua, Marinka Bulic, Yanwen Jiang, Matteo Carrara, Raffaele A. Calogero, William L. Kath, Neil L. Kelleher, Ji-Ping Wang, Olivier Elemento, Jonathan D. Licht

**Affiliations:** 1 Division of Hematology/Oncology, Northwestern University Feinberg School of Medicine, Chicago, Illinois, United States of America; 2 Arthritis and Tissue Degeneration Program and the David Z. Rosensweig Genomics Research Center, Hospital for Special Surgery, New York, New York, United States of America; 3 Biological Sciences Department, New York City College of Technology, City University of New York, Brooklyn, New York, New York, United States of America; 4 Department of Statistics, Northwestern University, Evanston, Illinois, United States of America; 5 Department of Chemistry and Molecular Biosciences, Chemistry of Life Processes Institute, Northwestern University, Evanston, Illinois, United States of America; 6 HRH Prince Alwaleed Bin Talal Bin Abdulaziz Alsaud Institute for Computational Biomedicine and Department of Physiology and Biophysics, Weill Cornell Medical College, New York, New York, United States of America; 7 Molecular Biotechnology Center, Department of Biotechnology and Health Sciences, University of Torino, Torino, Italy; 8 Department of Engineering Sciences and Applied Mathematics, Northwestern University, Evanston, Illinois, United States of America; Oxford University, United Kingdom

## Abstract

Overexpression of the histone methyltransferase MMSET in t(4;14)+ multiple myeloma patients is believed to be the driving factor in the pathogenesis of this subtype of myeloma. MMSET catalyzes dimethylation of lysine 36 on histone H3 (H3K36me2), and its overexpression causes a global increase in H3K36me2, redistributing this mark in a broad, elevated level across the genome. Here, we demonstrate that an increased level of MMSET also induces a global reduction of lysine 27 trimethylation on histone H3 (H3K27me3). Despite the net decrease in H3K27 methylation, specific genomic loci exhibit enhanced recruitment of the EZH2 histone methyltransferase and become hypermethylated on this residue. These effects likely contribute to the myeloma phenotype since MMSET-overexpressing cells displayed increased sensitivity to EZH2 inhibition. Furthermore, we demonstrate that such MMSET-mediated epigenetic changes require a number of functional domains within the protein, including PHD domains that mediate MMSET recruitment to chromatin. In vivo, targeting of *MMSET* by an inducible shRNA reversed histone methylation changes and led to regression of established tumors in athymic mice. Together, our work elucidates previously unrecognized interplay between MMSET and EZH2 in myeloma oncogenesis and identifies domains to be considered when designing inhibitors of MMSET function.

## Author Summary

Precise spatial and temporal gene expression is required for normal development, and aberrant regulation of gene expression is a common factor in many diseases, including cancer. Histone modifications contribute to the control of gene expression by altering chromatin structure and affecting the recruitment of transcriptional regulators. In this study, we demonstrate interplay between two oncogenic proteins, MMSET and EZH2, known to methylate histone H3 on lysine 36 (H3K36) and lysine 27 (H3K27), respectively. Overexpression of MMSET in myeloma cells increases global levels of H3K36 methylation, alters its normal distribution throughout the genome and decreases global levels of H3K27 methylation. We found that while the majority of the genome loses H3K27 methylation in the presence of MMSET, certain loci have augmented recruitment of EZH2 and enhanced H3K27 methylation, leading to transcriptional repression. Repression of these genes likely plays an important role in the disease because MMSET-overexpressing cells show higher sensitivity to small molecule inhibitors targeting EZH2-mediated methylation. Thus, our study suggests that the specific local changes may outweigh the gross global changes we frequently observe in cancer and implicates EZH2 as a novel therapeutic target in myeloma cells.

## Introduction

Epigenetic control of gene expression plays a critical role in many biological processes and aberrant chromatin regulation is the driving factor in a multitude of diseases, including cancer. Through studies of chromosomal rearrangements, copy number changes, and more recently, sequencing of cancer genomes, it has become apparent that genetic alterations of enzymes responsible for covalent modification of histones or DNA, including histone methyltransferases (HMTs), are a recurrent theme in the pathogenesis of malignancy. Recently, HMTs have attracted particular interest due to their potential as therapeutic targets [1], but our understanding of the mechanisms by which abnormal histone methylation leads to disease development is still incomplete.

The specificity of each HMT is encoded within the catalytic SET (Suppressor of variegation, Enhancer of zeste and Trithorax) domain. For example, trimethylation of lysine 27 on histone H3 (H3K27me3) is mediated by the EZH2 protein, a member of the Polycomb Repressive Complex 2 (PRC2) [2]. Binding of EZH2 and the presence of the H3K27me3 mark are found at transcriptionally repressed loci and have been shown to play a role in recruitment of additional transcriptional repressors, including DNA methyltransferases (DNMTs) [3], [4]. EZH2 gain-of-function mutations that enhance H3K27me3 levels are pathogenic for germinal center type large B cell lymphomas [5], [6], whereas global loss of EZH2 function due to mutation/deletion of *EZH2* or associated factors such as *SUZ12*, *EED* and *ASXL1* are associated with myeloid neoplasms [7]–[9].

MMSET (WHSC1/NSD2) is a histone methyltransferase whose enzymatic specificity in vivo is towards dimethylation of lysine 36 on histone H3 (H3K36me2) [10]–[12], an epigenetic mark associated with transcriptionally active loci [13]. Heterozygous deletions of MMSET are implicated in the developmental disorder Wolf-Hirschhorn syndrome (WHS), characterized by cognitive and developmental defects [14]. Similar phenotypic defects are observed in *MMSET*-deficient mice [15]. Alterations in MMSET expression are also linked to cancer. This was first described in multiple myeloma (MM), where ∼20% of cases overexpress *MMSET* due to the translocation t(4;14) [16], which places the *MMSET* and *FGFR3* loci under regulation of strong immunoglobulin enhancers, leading to abnormally high levels of these factors [17]. However, in 30% of cases, FGFR3 expression is not affected, suggesting that misregulation of MMSET may be the driving lesion of the disease [18], [19]. A growing body of literature demonstrates that increased expression of MMSET is associated with advanced stage solid tumors, including prostate, bladder, lung and skin cancers, where it may control oncogenic properties such as the epithelial-mesenchymal transition [20]–[23]. Furthermore, we recently identified a recurrent gain-of-function mutation of MMSET (E1099K) most commonly found in lymphoid malignancies, which enhances its methyltransferase activity and may functionally mimic overexpression seen in other cancers [24], [25]. The epigenetic alterations and biological consequences of MMSET overexpression in cancer are beginning to be elucidated. We and others showed that downregulation of MMSET expression in t(4;14)+ cell lines leads to decreased proliferation and loss of clonogenic potential [12], [26]. In myeloma and prostate cells, overexpression of MMSET causes a dramatic global increase in H3K36me2, accompanied with a concomitant genome-wide loss of H3K27me3 [12], [20], [27]. The change in histone methylation is dependent on the HMT activity of MMSET and leads to altered chromatin structure and aberrant gene expression [12].

In this work, we aimed to elucidate the mechanisms by which MMSET alters gene expression in MM. Genome-wide chromatin analysis showed that MMSET overexpression led to a widespread redistribution in H3K36me2 across promoters, gene bodies and intergenic regions, and gene activation correlated with removal of the inhibitory H3K27me3 chromatin mark. Surprisingly, overexpression of MMSET induced transcriptional repression at specific loci that became highly enriched for EZH2 and H3K27me3. This increase was associated with augmented sensitivity to small molecule inhibitors targeting EZH2 methyltransferase activity. The ability of an epigenetic regulator to modify histones or DNA depends on its ability to target specific loci through direct interaction with chromatin, or through recruitment by other transcriptional cofactors. We identified the domains of MMSET that are required for its recruitment to chromatin and that are necessary for methylation of H3K36 and loss of H3K27 methylation in t(4;14)+ cells. Both of these functions are necessary for the oncogenic potential of MMSET. Lastly, we validated MMSET as a therapeutic target by showing that loss of MMSET expression in established t(4;14)+ tumors led to a decrease in tumor burden and an increase in survival. Together, our results reveal an interplay between H3K36 and H3K27 methylation in t(4;14)+ myeloma and identify the domains of MMSET that could be targeted in efforts to improve outcomes of this currently incurable disease.

## Results

### MMSET alters the epigenetic landscape of t(4;14)+ myeloma cells

**Figure 1:**
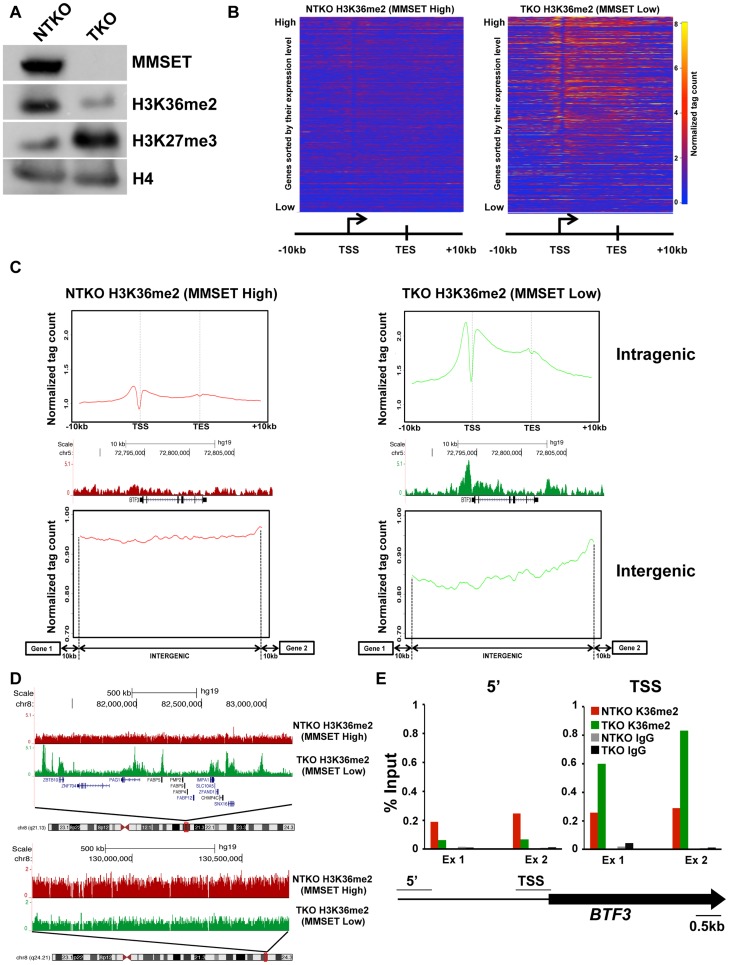
MMSET overexpression alters normal H3K36me2 distribution. (A) Loss of MMSET expression in t(4;14)+ cells depletes H3K36 methylation and increases H3K27 methylation. Immunoblot of nuclear extracts from t(4;14)+ KMS11 cells with inactivated translocated (TKO) or wild-type (NTKO) *MMSET* allele were probed using antibodies for MMSET, H3K36me2, H3K27me3 and total H4 as control. (B) Heatmaps of H3K36me2 distribution between NTKO (left) and TKO (right) cells. On the y-axis, genes were ranked based on their expression level from high (top) to low (bottom) and on the x-axis, normalized read density from ChIP-seq experiments was plotted in gene bodies and 10 kb upstream and downstream from the transcription start (TSS) and end (TES) sites. (C) MMSET alters H3K36me2 distribution in intragenic (top) and intergenic (bottom) regions. (Top) Average read density across 15,386 genes in NTKO (left, red) and TKO (right, green) cells. (Middle) UCSC genome browser view of the H3K36me2 distribution on a representative locus (*BTF3*) in NTKO and TKO cells. (Bottom) Average normalized tag density of H3K36me2 in 6,172 intergenic regions in NTKO (left) and TKO (right) cells. (D) Representative UCSC genome browser view of the H3K36me2 distribution on chromosome 8 of NTKO (red) and TKO (green) cells. Top panel encompasses a gene-rich region of the chromosome and the bottom insert represents a 1MB region of the 8q24 gene desert. (E) ChIP-qPCR for H3K36me2 on the *BTF3* locus. Methylation enrichment was tested 5 kb upstream of the TSS (5′) and at the TSS (right). Two independent biological replicates are shown.

We and others have reported that the overexpression of MMSET in t(4;14)+ myeloma cells increases global levels of H3K36 dimethylation [11], [12]. To investigate how the pattern of H3K36me2 genomic distribution is affected by MMSET abundance, we performed chromatin immunoprecipitation followed by high-throughput sequencing (ChIP-seq) in TKO and NTKO cells (two independent biological replicates for each sample). TKO is a t(4;14)+ KMS11 cell line in which the rearranged *IGH-MMSET* allele has been inactivated by homologous recombination. These cells express only one wild-type copy of the *MMSET* gene at physiological levels yielding low basal levels of H3K36me2 (Figure 1A) [26]. In their counterpart, NTKO cells, the wild-type *MMSET* allele is inactivated, but high levels of MMSET and H3K36me2 are maintained from the remaining rearranged *IGH-MMSET* allele (Figure 1A). We performed microarray analysis in NTKO and TKO cells to obtain global expression levels in these cells. In agreement with previous studies [11], in MMSET-low TKO cells, the presence of H3K36me2 positively correlated with highly expressed genes (Figure 1B; Supplemental Figures S1A, S1B). Typically, the enrichment of H3K36me2 peaked just upstream of the transcription start site (TSS) and decreased towards the 3′ end of a gene (Figures 1B, 1C; Supplemental Figure S1B). Our ChIP-seq analysis of NTKO cells revealed that this characteristic distribution of H3K36me2 was disrupted in MMSET-high conditions. Despite very high global levels of H3K36 dimethylation in NTKO cells, H3K36me2 enrichment did not localize to specific loci or domains (Figures 1B and 1C intragenic). Instead, H3K36me2 enrichment was dispersed more evenly throughout the genome and lacked clear boundaries (Figure 1C intergenic, 1D bottom). In MMSET-high NTKO cells characteristic peaks of H3K36me2 adjacent to the TSS were eliminated and a lower uniform level of H3K36me2 was measured throughout gene bodies (Figure 1C intragenic; Supplemental Figure S1B). This decrease of intragenic H3K36me2 seemed paradoxical as immunoblot and mass spectrometry clearly demonstrated an approximately 8-fold increase of H3K36me2 in NTKO cells (Figure 1A) [12], [27]. This inconsistency was resolved by examining H3K36me2 within intergenic regions, which comprise 97% of the genome compared to the 3% of the genome that is protein-coding [28]. Plotting the density of sequence tags across 6,172 intergenic regions and comparing the relative H3K36me2 enrichment revealed that MMSET-high NTKO cells have an increase in abundance of the H3K36me2 modification (Figure 1C). A large scale view over a gene-rich region illustrates that peaks of H3K36me2 are obliterated in MMSET-high NTKO cells (Figure 1D, top), whereas generally low levels of H3K36me2 in a gene-poor region are increased in the presence of high levels of MMSET (Figure 1D, bottom; Supplemental Figure S1B). To confirm that these findings were not due to the sequencing biases that may occur due to dramatically different levels of H3K36me2 in the two cell types, we performed ChIP-qPCR analysis on three loci, *BTF3, SNX16* and *GAPDH*, whose expression does not change in response to altered levels of MMSET. In MMSET-high NTKO cells, H3K36me2 enrichment remains relatively constant at the promoter or upstream of *BTF3, SNX16* and *GAPDH* TSS (Figure 1E, Supplemental Figures S2A and S2B). By contrast, in MMSET-low TKO cells H3K36me2 peaks around the TSS and then drops dramatically in regions away from the promoter (Figure 1E, Supplemental Figures S2A and B). These data confirm that the typical peaks of H3K36me2 enrichment at the transcriptionally active loci are completely lost due to MMSET overexpression.

**Figure 2:**
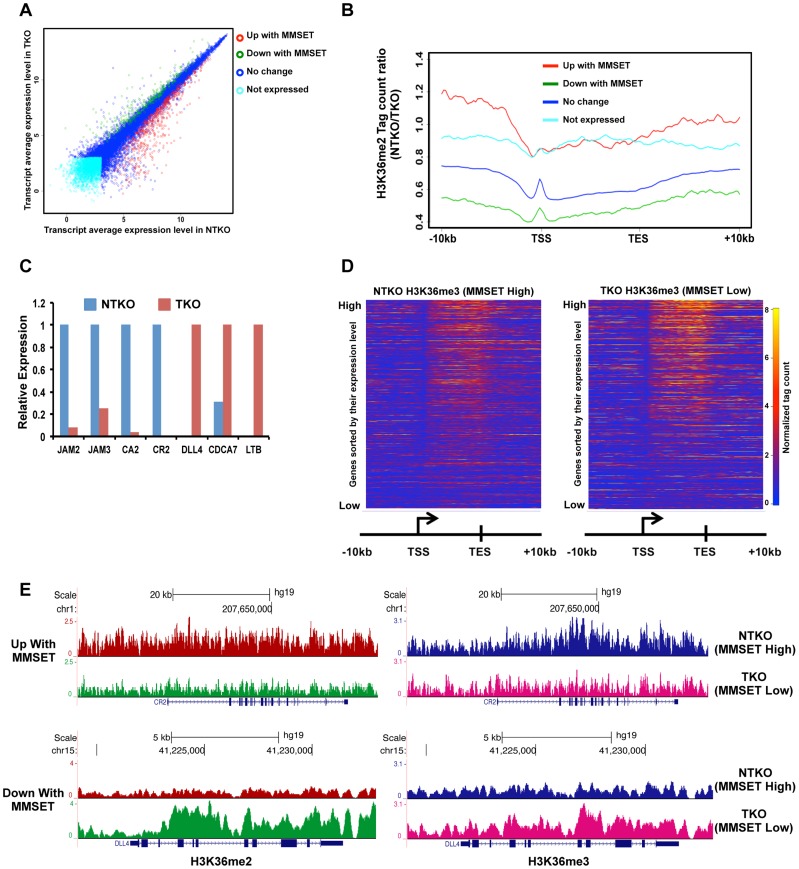
High levels of MMSET lead to altered gene expression. (A) Dot plot representing differentially expressed genes in NTKO and TKO cells from the microarray data. Genes upregulated and downregulated by MMSET (p<0.002) are in red (522 genes) and green (308 genes), respectively; genes that do not change expression are in dark blue (10,884 genes) and genes that are not expressed (2,234 genes) in either cell line are light blue. (B) Tag density profile of H3K36me2 distribution across different gene groups from A. Ratio between the number of reads in NTKO and TKO cells is presented on the y-axis. (C) Quantitative RT-PCR validation of several genes upregulated and downregulated by MMSET overexpression. (D) Heatmaps of H3K36me3 distribution between NTKO (left) and TKO (right) cells. Data were plotted as in Figure 1B. (E) Density tracks for H3K36me2 and H3K36me3 on a gene upregulated by MMSET (*CR2* - upper panel) and a gene downregulated by MMSET overexpression (*DLL4* – lower panel). H3K36me2 tracks are in red and green for NTKO and TKO cells, respectively; H3K36me3 tracks are in blue (NTKO) and fuchsia (TKO).

Despite the wide-ranging increase of H3K36me2 levels in NTKO cells, we found that MMSET affects expression of only a specific subset of genes. Gene expression profiling identified 522 genes upregulated with overexpression of MMSET and 308 genes that are repressed in MMSET-high NTKO cells (Figure 2A; Supplemental Table S1). To better understand the basis of expression changes associated with MMSET abundance, we examined the compiled H3K36me2 ChIP-seq profiles of genes sorted based on their expression pattern in NTKO and TKO cells. Specifically, regions upstream of the TSS of genes activated in the presence of MMSET are more enriched for H3K36me2 in NTKO than TKO cells (Figure 2B, red line and Supplemental Figure S3A). These include adhesion molecules such as *JAM2* and *JAM3* as well as *CR2* (Figure 2C), the latter of which was shown to play a role in the interaction of myeloma cells with the bone marrow stroma [29]. However, within gene bodies of genes activated by MMSET, the levels of H3K36me2 were very comparable between NTKO and TKO cells (Figure 2B, red line). This result suggested that the action of MMSET at the promoters was important in regulation of these genes. Genes that were not expressed in either NTKO or TKO cells did not have any significant changes in the density of H3K36me2 (Figure 2B, light blue line). However, genes repressed in the presence of MMSET (Figure 2B, green line) or genes whose expression is not altered due to MMSET (Figure 2B, dark blue line) seemed to be protected from the global increase in H3K36me2 in NTKO cells and were found to have higher levels of H3K36me2 in MMSET-low TKO cells. We also performed ChIP-seq for trimethylated H3K36 (H3K36me3) in NTKO and TKO cells, a chromatin mark enriched in gene bodies of highly expressed genes [30]. Although global H3K36me3 distribution patterns were similar between NTKO and TKO cells (Figure 2D; Supplemental Figure S3B), as expected, the subset of genes upregulated in MMSET-high NTKO cells exhibited elevated enrichment of H3K36me3 throughout their intragenic regions (Figure 2E). Similarly, genes downregulated in the presence of high levels of MMSET, such as *DLL4*, correlated with increased levels of H3K36me2 and H3K36me3 in MMSET-low TKO cells (Figures 2C and 2E).

**Figure 3:**
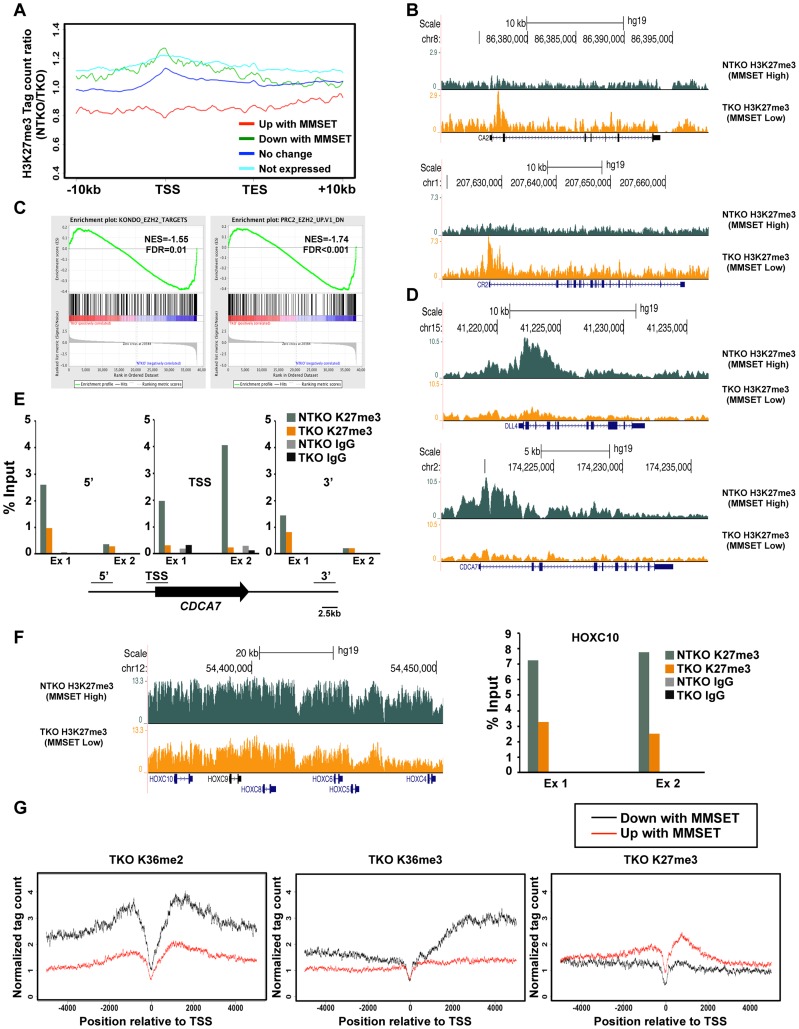
MMSET alters genome-wide patterns of H3K27me3 methylation. (A) Tag density profile of H3K27me3 distribution across different gene groups from Figure 2A. The ratio between read numbers in NTKO and TKO cells is presented on the y-axis. (B) UCSC genome browser display of H3K27me3 density tracks surrounding the transcription start site of two MMSET activated genes, *CA2* (top) and *CR2* (bottom). (C) GSEA analysis of genes upregulated by MMSET shows enrichment of previously identified EZH2 target genes. (D) UCSC genome browser display of H3K27me3 density tracks surrounding the transcription start site of two MMSET repressed genes, *DLL4* (top) and *CDCA7* (bottom). (E) ChIP-qPCR for H3K27me3 on *CDCA7* locus. Methylation enrichment was tested on the promoter (TSS) and on the regions upstream (5′) and downstream (3′) from the TSS. Two independent biological replicates are shown. (F) UCSC genome browser of H3K27me3 enrichment on non-expressed genes of the *HOXC* cluster (left) and ChIP-qPCR for H3K27me3 on the *HOXC10* locus (right). Two independent biological replicates are shown. (G) Tag density profile of H3K36me2 (left), H3K36me3 (middle) and H3K27me3 (right) distribution of differentially expressed genes in TKO cells.

The relatively high number of repressed genes in NTKO cells was unexpected given the global chromatin effects, increased H3K36me2 and decreased H3K27me3, associated with MMSET overexpression (Figure 1A). ChIP-seq analysis of H3K27me3 in NTKO and TKO cells revealed that the upregulation of gene expression in the presence of MMSET was accompanied by a loss of H3K27me3, particularly in regions 5′ to the TSS (Figures 3A, red line, Figure 3B and Supplemental Figure S3A). Gene Set Enrichment Analysis (GSEA) showed that the subset of genes upregulated in MMSET-high NTKO cells represent direct targets of EZH2 and H3K27 methylation (Figure 3C) [31]–[33]. This suggests that MMSET itself or its resultant H3K36me2 modification may prevent PRC2 binding to these loci, thereby inducing gene expression by relief of active Polycomb repression. By contrast, genes with expression levels that were unaffected by MMSET abundance showed no significant difference in H3K27me3 enrichment despite the global decrease of H3K27me3 in NTKO cells (Figure 3A, dark blue). However, genes repressed in the presence of high levels of MMSET or transcriptionally silent in both cell types showed an increased abundance of H3K27me3 in MMSET-high NTKO cells (Figure 3A, green, light blue). On many genes repressed in the presence of MMSET a large increase of H3K27me3 near the start site of transcription was observed in NTKO cells (Figure 3D, 3E and Supplemental Figures S4A and S4B). The *HOXC* cluster, whose expression could not be detected in this cell system, demonstrated fairly high levels of H3K27me3 dispersed across a >60 kb genomic segment in MMSET-low TKO cells, that was increased ∼2.5-fold in MMSET-high NTKO cells (Figure 3F). The chromatin landscape of genes exhibiting decreased expression in MMSET-high cells was examined in TKO cells. Here, genes that were repressed in the presence of MMSET and activated in TKO cells had increased levels of H3K36me2 and H3K36me3 and decreased levels of H3K27me3 (Figure 3G), and included known EZH2/PRC2 targets, such as *DLL4* and *CDCA7* (Figure 3D) [32], [34]. Thus, overexpression of MMSET not only modifies H3K36me2 distribution, but also leads to a drastic change in the distribution pattern of the repressive H3K27me3 mark. Whereas MMSET-overexpressing myeloma cells show a genome-wide decrease in H3K27me3, specific loci are able to maintain and even gain higher levels of H3K27 methylation in the presence of MMSET, leading to transcriptional repression.

### MMSET overexpression alters patterns of EZH2 binding

Our previous work demonstrated that the epigenetic landscape in t(4;14)+ cells is established by a competition between the methylation activities of EZH2 and MMSET for the histone H3 tail substrate [27]. Furthermore, active chromatin marks, including H3K36me2, were shown to inhibit PRC2 from both binding the nucleosomes and methylating the histones [35], [36]. Kinetic studies revealed that once histone H3 reaches the dimethylation state at H3K36, the effective rates of H3K27 di- and trimethylation on the same histone molecule drop dramatically [27]. Furthermore, we showed that MMSET-high cells have increased rates of H3K27 demethylation contributing to the global loss of this modification [27]. These data suggest that the global decrease of H3K27 methylation in the presence of MMSET may be due to the inability of EZH2 and the PRC2 complex to bind chromatin. If this is true, then an increased concentration of unbound EZH2 would be available to bind genomic regions that are able to maintain H3K27me3 in the presence of high levels of MMSET. Therefore, this increased ratio of free enzyme to available substrate could be responsible for the enhanced enrichment of H3K27 trimethylation at specific loci in NTKO cells.

**Figure 4:**
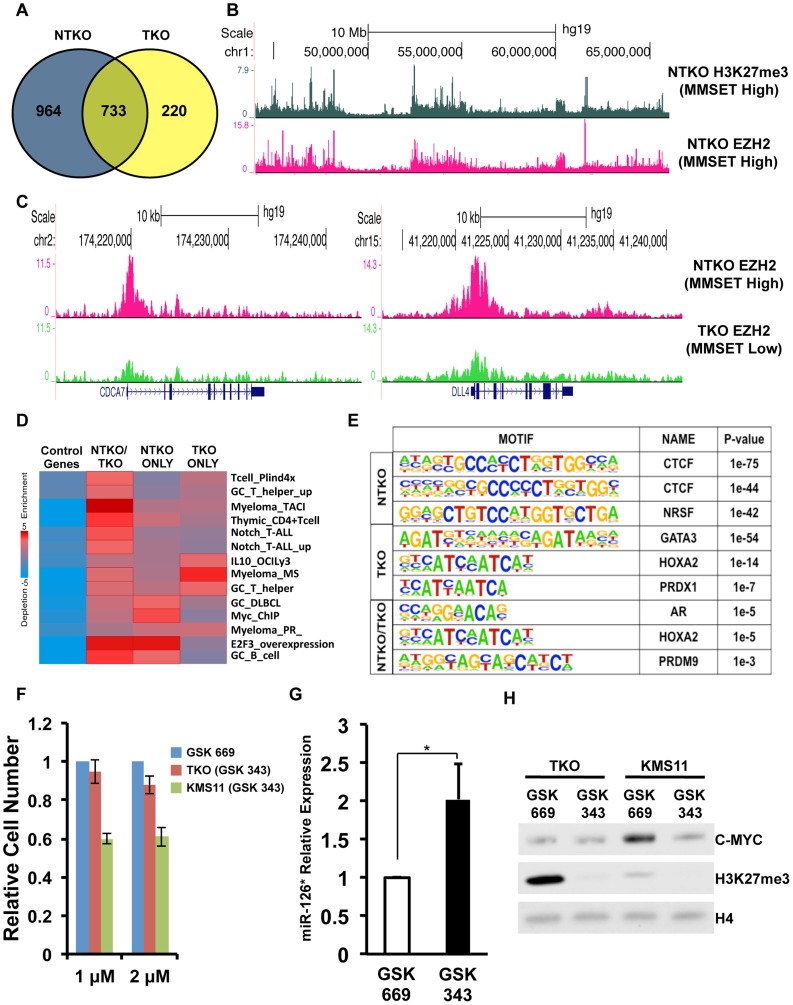
MMSET alters EZH2 binding in t(4;14)+ myeloma cells. (A) Venn diagram showing overlap of genes bound at their promoters by EZH2 in NTKO (blue) and TKO (yellow) cells. (B) UCSC genome browser display of H3K27me3 (top, gray) and EZH2 binding (bottom, red) in NTKO cells. (C) UCSC genome browser display of EZH2 ChIP-seq tracks in NTKO (top, red) and TKO (bottom, green) cells associated with MMSET-repressed genes, *CDCA7* (left) and *DLL4* (right). (D) Heat map of over-represented gene categories among genes bound by EZH2 in either NTKO cells, TKO cells or both cell types. Enrichment was measured using iPAGE analysis [74]. (E) Motif analysis using HOMER [73] identified conserved sequences bound by EZH2 in NTKO cells, TKO cells or both cell types. (F) Relative cell number of MMSET-high and MMSET-low cells treated with indicated doses of the EZH2 small molecule inhibitor (GSK343). Inactive compound, GSK669, was used as a control. Graph represents four independent experiments +/− standard deviation. (G) Quantitative RT-PCR measurement of miR-126* expression in KMS11 cells treated with GSK669 or GSK 343. Graph represents average expression from three independent experiments +/− standard deviation (* p<0.05). (H) Representative immunoblot of nuclear extracts from MMSET-high (KMS11) and MMSET-low (TKO) cells treated for seven days with 2 µM EZH2 inhibitor (GSK343) or inactive control (GSK669). This experiment was performed in biological triplicate.

To test this hypothesis, we examined EZH2 distribution in TKO and NTKO cells by ChIP-seq (two highly correlated independent biological replicates, r = 0.82 and r = 0.84, respectively) (Supplemental Figure S5A). This analysis identified 10,581 EZH2 peaks (associated with the promoters of 1,697 genes) in the MMSET-high NTKO cells and 5,516 (953 genes) in the TKO cells (Figure 4A and Supplemental Table S2). Of these genes, 733 were common between the two cell types and 964 genes had enriched EZH2 binding exclusively in the NTKO cells. As predicted, EZH2 peaks that are shared between NTKO and TKO cells are on average higher and broader in NTKO cells (Supplemental Figure S5B). In MMSET-high cells, enhanced localization of EZH2 closely tracked with H3K27me3 enrichment (Figure 4B), including *DLL4* and *CDCA7* promoters (Figure 4C). Analysis of genes bound by EZH2 only in MMSET-high NTKO cells, using a library of lymphoid biology gene expression signatures [37], showed that they included genes known to play a role in normal germinal center B cells (GC_B_cell category), as well as known B cell MYC targets (Myc_ChIP category) (Figure 4D and Supplemental Table S3). Thus, aberrant EZH2-mediated repression of genes known to play a role in lymphoid biology may be important for MMSET-induced oncogenesis. Loci bound by EZH2 only in TKO cells were enriched for genes found to be upregulated in t(4;14)+ patient samples (Figure 4D, Myeloma_MS category), suggesting that MMSET overexpression reverses normal silencing of these genes by the PRC2 complex.

Considering that many EZH2 bound regions were unique to either MMSET-high or MMSET-low cells, we examined the underlying sequence to determine if specific transcription factor motifs were over-represented within these regions. This analysis revealed that regions bound by EZH2 exclusively in MMSET-low TKO cells coincided with GATA3, HOXA2 and PDX1 motifs (Figure 4E). In breast cancer cells, GATA3 and EZH2 are functionally antagonistic, suggesting that similar interplay between these two factors may also exist in myeloma [38]. Interestingly, EZH2-bound regions specific to MMSET-high NTKO cells were associated with DNA motifs that resemble known CTCF DNA binding sites (Figure 4E), implying a possible mechanism where insulator sequences may protect these loci from methylation by MMSET. Additional DNA motifs included poly-G and poly-C-rich sequences (Supplemental Figure S5C), resembling PRC2 recruitment motifs defined in ES cells [39]. To determine whether the enhanced binding of EZH2 may play a functional role in myeloma cell survival, we treated MMSET-high and MMSET-low cells with recently described small molecule inhibitor of EZH2 [40]. Indeed, MMSET-high cells were more sensitive to EZH2 inhibition (Figures 4F; Supplemental Figures S5D and S6A), suggesting that some of the newly acquired EZH2 binding sites in MMSET-high cells are critical for survival of these cells.

Translocations of c-*MYC* are common in multiple myeloma and the MYC activation signature is observed in a majority of MM patient samples [41], [42]. Our previous study implicated MMSET overexpression in the regulation of MYC through downregulation of a microRNA, miR-126* [43]. In MMSET-overexpressing cells expression of miR-126* levels is decreased through recruitment of transcriptional repressors such as KAP1. Our ChIP-seq analysis shows that MMSET overexpression leads to EZH2 and H3K27me3 accumulation upstream of the miR-126* locus (Supplemental Figure S6B). Upon treatment with EZH2 inhibitors, MMSET-overexpressing cells increase expression of miR-126* (Figure 4G). In accordance with our previous study, increased miR-126* levels were associated with a dramatic downregulation of MYC (Figure 4H). By contrast, EZH2i had no effect on MYC levels in inhibitor-insensitive TKO cells (Figure 4H). As myeloma cells are frequently dependent on MYC for cell growth, the reduction in MYC levels in response to EZH2i likely contributes to observed decrease in proliferation of MMSET-overexpressing cells. To investigate other potential mechanisms EZH2 sensitivity of MMSET-overexpressing cells, we performed RNA-seq analysis in KMS11 and TKO cells treated for seven days. In accordance with its role in gene repression, inhibition of EZH2 primarily lead to activation of gene transcription: 561 genes were upregulated in MMSET-high cells and 1412 genes were activated in the TKO cells (Supplemental Table S4). However, only 165 genes were upregulated in both cell types further strengthening the idea that EZH2 regulates expression of different loci in the presence or absence of MMSET. Genes upregulated in MMSET-overexpressing cells include tumor suppressor DACH1 and members of the WNT signaling pathway, however the role of these genes in suppression of proliferation remains to be elucidated. Interestingly, EZH2 inhibition in the TKO cells activated a number of genes identified to be upregulated in t(4;14)+ myeloma patients (Myeloma_MS_subgroup_up) suggesting that loss of H3K27 methylation, through MMSET overexpression or EZH2i, can activate similar gene sets (Supplemental Figure S6C; Supplemental Table S5). This supports our notion that MMSET activates expression by preventing EZH2 activity at specific loci due to the mutually opposing interplay of H3K36 and H3K27 methylation. Together, we conclude that MMSET overexpression alters the genomic organization of EZH2 across the myeloma genome and this effect, similar to other cancers, induces misregulation of specific Polycomb target genes that contribute to pathogenesis.

### Oncogenic MMSET function depends on its PHD and PWWP domains

**Figure 5:**
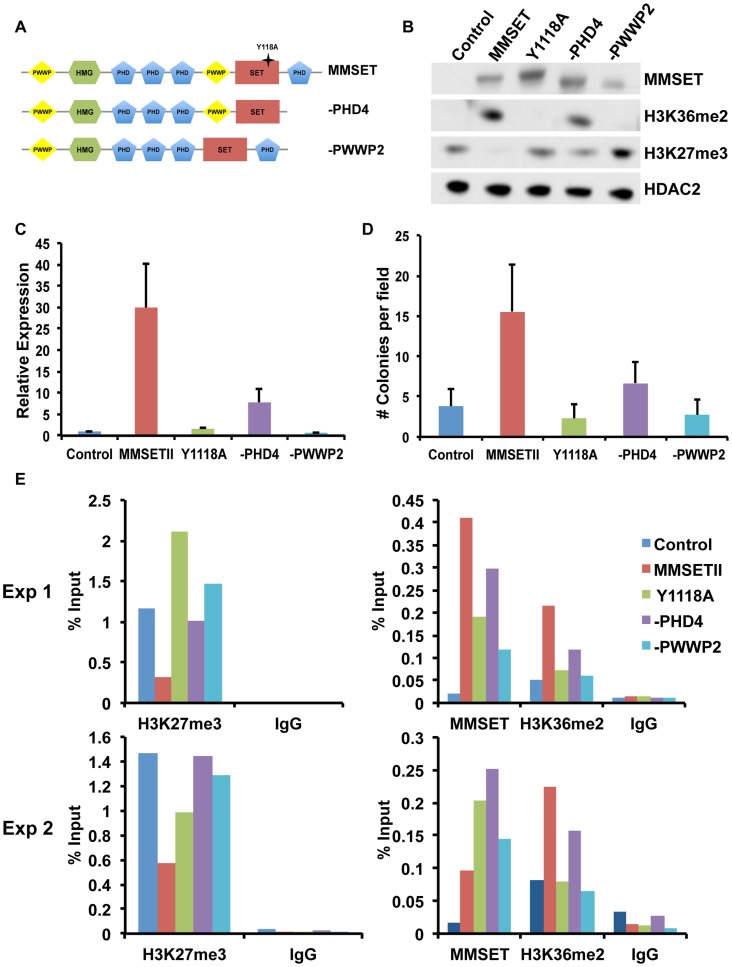
The MMSET-induced epigenetic switch depends on multiple domains. (A) Diagram of the wild-type (amino acids 1–1365) and deletion MMSET constructs used for repletion experiments. Star represents location of the Y1118A mutation. –PHD construct encompasses amino acids 1–1209 and –PWWP2 construct is missing amino acids 877–995. (B) Representative immunoblot of nuclear extracts from repleted TKO cells was probed with the indicated antibodies. At least two independent infections were performed for each construct. (C) Representative qRT-PCR for *JAM2* in repleted TKO cells. Measurements were performed from multiple infections in at least duplicate and the graph represents average expression +/− standard deviation. (D) Methylcellulose colony formation assay using repleted TKO cells. Experiment was performed in triplicate and at least six different fields were counted. Graph represents average colony count +/− standard deviation. (E) ChIP assay on the *JAM2* promoter using repleted TKO cell. Two independent biological replicates are shown.

In addition to the enzymatically active SET domain, MMSET possesses four PHD domains commonly implicated in chromatin binding [44]. To determine whether these and other conserved domains of MMSET are required for myelomagenesis, we repleted TKO cells with either wild-type MMSET or deletion mutants and assessed for changes in chromatin modifications, gene expression and growth (Figure 5A). Expression of wild-type MMSET in TKO cells re-established high levels of H3K36me2 and loss of H3K27me3 (Figure 5B), activated transcription of specific genes, such as *JAM2* (Figure 5C; Supplemental Figure S7A), stimulated proliferation (Supplemental Figure S7B) and increased colony formation (Figure 5D; Supplemental Figure S7C). A point mutation at tyrosine 1118 (Y1118A) that abrogates the HMT activity of MMSET [12] prevented the re-establishment of H3K36 and H3K27 methylation in vivo (Figure 5B; Supplemental Figure S7D) and failed to stimulate gene expression (Figure 5C), cell growth [12] and colony formation (Figure 5D; Supplemental Figure S7C). A construct missing the C-terminal portion of the protein, including PHD finger #4 (-PHD4), was able to methylate H3K36, albeit at lower levels (Figure 5B) [27], and resulted in an incomplete loss of H3K27 methylation (Figure 5B), yielding an intermediate alteration of gene expression (Figure 5C; Supplemental Figure S7A), growth stimulation (Supplemental Figure S7B) and colony formation (Figure 5D and Supplemental Figure S7B). These data suggest that the biological contribution of MMSET in myeloma cells not only depends on its ability to stimulate H3K36me2 levels, but also depends on the degree of inhibition of H3K27 methylation.

MMSET also contains two PWWP domains, the first of which has been suggested to nonspecifically bind chromatin [45]. However, in many MM cases, the t(4;14) breakpoint disrupts MMSET 3′ to the exons encoding PWWP1, leading to the overexpression of a truncated MMSET lacking this domain. Thus, while PWWP1 may play an important role for normal MMSET function, it likely does not contribute to oncogenesis. However, loss of the second PWWP domain rendered MMSET enzymatically inactive (Figure 5B), and this construct (-PWWP2) was unable to stimulate growth (Figure S5E), alter gene expression (Figure 5C; Supplemental Figure S7A) or promote colony formation (Figure 5D; Supplemental Figure S7C). Interestingly, all deleted or mutated constructs were still able to bind chromatin (Figure 5E). Nevertheless, both -PWWP2 and Y1118A mutants were unable to mediate methylation of lysine H3K36 on the *JAM2* locus and thus are unable to induce gene expression (Figure 5C; Supplemental Figure S7A). By contrast, -PHD4 expression led to H3K36 methylation but its inability to induce complete demethylation of H3K27 allowed for only partial *JAM2* activation (Figure 5C; Supplemental Figure S7A). We conclude that the ability of MMSET to induce a complete H3K36/H3K27 methylation switch in myeloma cells depends on a complex interplay of several domains of the protein. Furthermore, our data suggest that both methylation of H3K36me2 and demethylation of H3K27me3 are required for MMSET to fully alter gene expression observed in myeloma.

### MMSET binding to chromatin and methylation of histones depends on specific PHD domains

**Figure 6:**
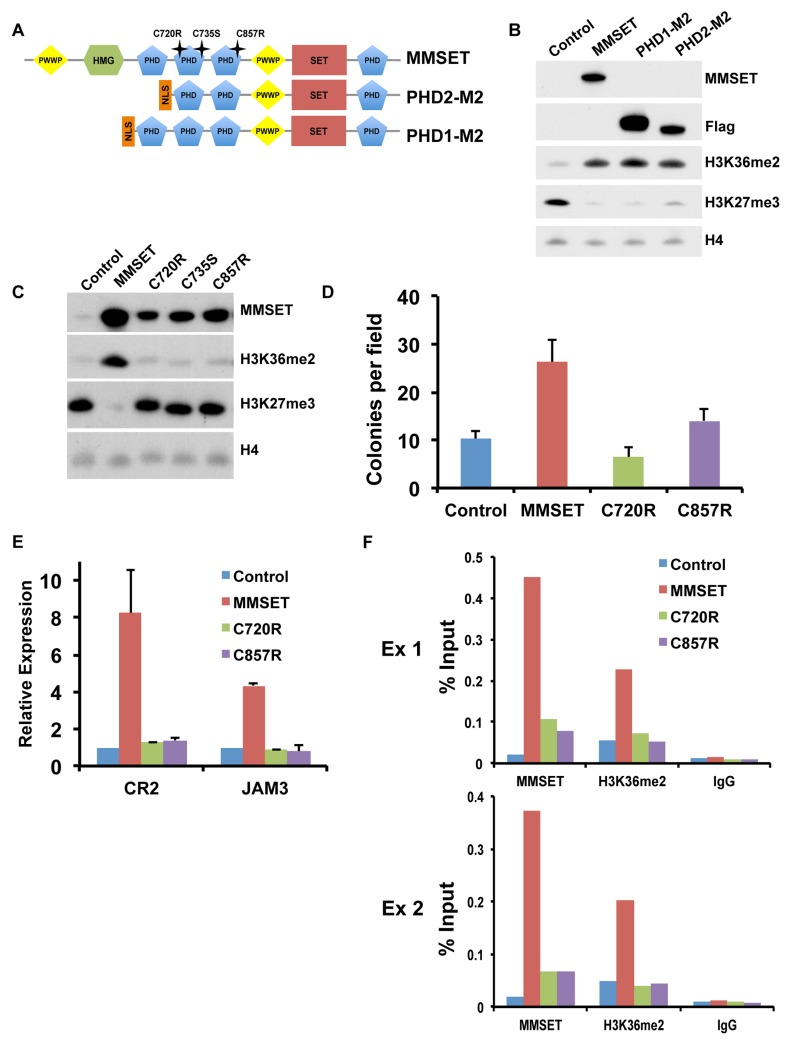
PHD fingers 2 and 3 are required for proper MMSET function. (A) Diagram of the MMSET constructs used. PHD2-M2 and PHD1-M2 constructs start at amino acid 712 and 657, respectively. Mutations in the PHD domains are indicated as stars above the full-length MMSET. (B) Representative immunoblot on nuclear extracts from repleted TKO cells probed with the indicated antibodies. At least two independent infections were performed for each construct. (C) Nuclear extracts from the TKO cells repleted with PHD mutants were immunoblotted with the indicated antibodies. At least two independent infections were performed for each construct. (D) Colony formation with TKO cells repleted with mutated PHD constructs. Experiment was performed in triplicate and at least six different fields were counted. Graph represents average colony count +/− standard deviation. (E) Quantitative RT-PCR of MMSET target genes *CR2* and *JAM3* using RNA from repleted TKO cells. Experiment was performed in duplicate and graph represents average gene expression +/− standard deviation. (F) Mutations in the PHD fingers prevent MMSET binding to chromatin. ChIP assay on the *JAM2* promoter using repleted TKO cells. Two independent biological replicates are shown.

We showed previously that the MMSET C-terminal isoform, REIIBP, which contains the third and fourth PHD fingers, the second PWWP domain and the SET domain, is not able to methylate histones in TKO cells [12]. We systematically added back additional domains of MMSET to REIIBP and found that addition of PHD finger 2 or PHD fingers 1 and 2 together (Figure 6A) induced methylation of H3K36 and demethylation of H3K27me3 (Figure 6B), as well as enhanced colony formation (Supplemental Figure S8A). Mutations and deletions in *NSD1*, an HMT closely related to MMSET, are implicated in Sotos syndrome, a disorder characterized by developmental overgrowth and cognitive disabilities [46]. We mapped previously identified mutations in NSD1 from Sotos syndrome patient samples to MMSET in attempt to identify important domains that are required for proper function of the two proteins (Figure 6A). Single point mutations of cysteine residues 720, 735 or 857, all within the second or third PHD finger of MMSET, rendered MMSET incapable of modulating H3K36 and H3K27 levels (Figure 6C). Similar to the enzymatically-dead SET domain mutant, expression of these PHD point mutants in TKO cells failed to stimulate colony formation (Figure 6D; Supplemental Figure S8B) or activate gene expression (Figure 6E). Importantly, ChIP assays for MMSET in repleted TKO cells revealed that the C720R and C857R mutant proteins exhibited dramatically reduced binding to the *JAM2* promoter, likely explaining their failure to methylate histones (Figure 6F). These data suggest that the PHD fingers of MMSET play an important role in recruitment of the protein to chromatin. In addition, these findings suggest that the Sotos syndrome mutations in NSD1 may have similar consequences, rendering the enzyme incapable of regulating chromatin structure and gene expression.

### Targeting MMSET decreases tumor burden in NOD/SCID mice

**Figure 7:**
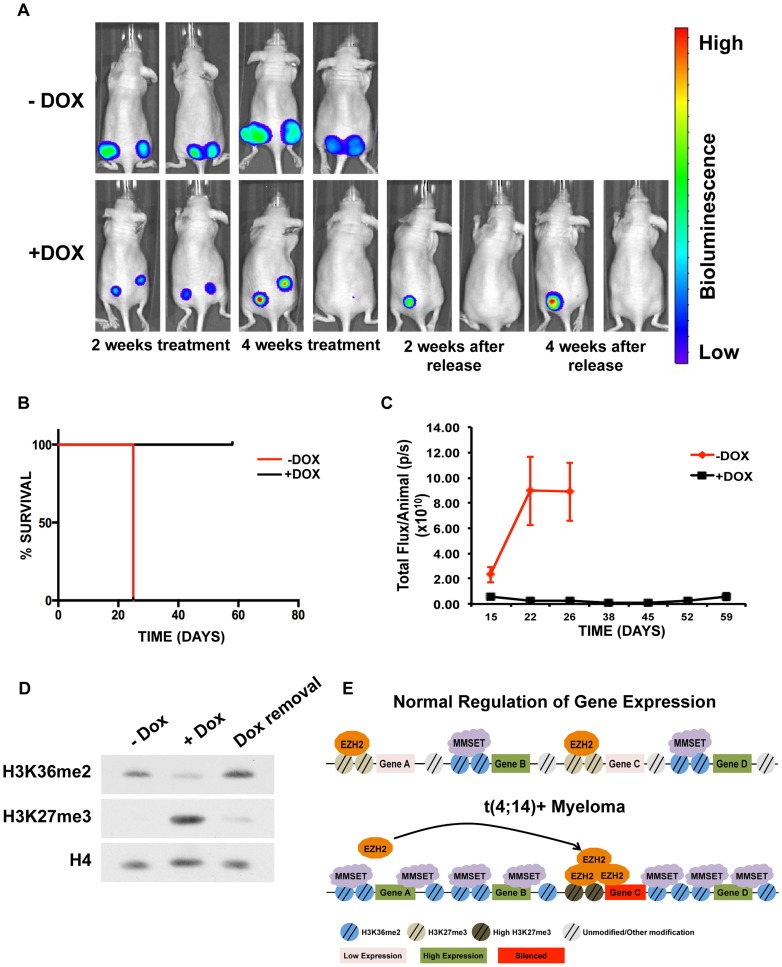
Targeting MMSET in t(4;14)+ tumors prolongs survival. (A) Mouse xenograft model using t(4;14)+ KMS11 cells harboring a luciferase gene and a doxycycline-inducible MMSET-specific shRNA. Animals that were not administered doxycycline (−Dox) are shown on the top and animals that were given doxycycline are shown on the bottom (+Dox). Two representative animals are shown from each group (n = 5) at the following timepoints: 2 weeks after treatment; 4 weeks after treatment; 2 weeks after release from Dox; and 4 weeks after release from Dox. –Dox animals were sacrificed 26 days after treatment initiation due to tumor size. The same two animals are shown at each time points. (B) Kaplan-Meier curve of the xenograft mouse experiment from A. (C) Measurement of the luciferin signal in treated (black) and untreated (red) mice over time. (D) Immunoblot for H3K36me2 and H3K27me3 using nuclear extracts from tumors isolated from the untreated or doxycycline-treated mice (E) Proposed model for explaining how MMSET overexpression alters the epigenetic landscape of myeloma cells. Methylation of H3K36 by MMSET induces a global decrease of H3K27me3, leading to activation of gene expression (bottom). Additionally, an overabundance of K36 methylation alters genome-wide EZH2 binding, inducing focal increases in H3K27 methylation and gene repression.

Elevated expression of MMSET in a number of different types of cancer suggests that inhibiting MMSET may be therapeutically advantageous beyond multiple myeloma. However, because MMSET translocation in myeloma occurs early in the premalignant MGUS (Monoclonal Gammopathy of Undetermined Significance) stage of the disease, it is unclear to what extent fully developed tumors depend on MMSET expression or whether targeting MMSET can lead to tumor reduction. To test whether MMSET reduction can inhibit myeloma growth in vivo, we injected the flanks of NOD/SCID mice with t(4;14)+ KMS11 cells expressing a doxycycline-inducible shRNA targeting *MMSET*. We previously demonstrated that expression of this shRNA decreases MMSET and H3K36me2 levels, increases H3K27me3 levels and leads to cell growth arrest [12]. Expression of the luciferin gene in the KMS11 cells allowed for in vivo live-cell imaging to monitor disease development. Tumors were allowed to grow for seven days, after which half of the mice were given doxycycline (dox) in their water to induce shRNA expression. As a result, all of the treated animals had dramatically reduced tumor volumes and in some cases, complete regression (Figures 7A–C and Supplemental Figure S9). By contrast, five weeks after injection of tumor cells, all untreated animals required sacrifice due to tumor progression. The reduction in tumor size in dox-treated animals was accompanied with a global loss of H3K36 dimethylation and an increase in H3K27 trimethylation (Figure 7D). To determine whether this was a long-lasting effect, we removed doxycycline after four weeks of treatment and observed the animals for four additional weeks. Even in the absence of shRNA expression, some tumors continued to decrease in size (Figure 7A). Animals whose tumors disappeared completely remained tumor-free even in the absence of doxycycline. However, tumors that persisted during induction of the *MMSET* shRNA eventually started to grow back upon doxycycline removal, albeit at a reduced rate. Thus, we conclude that established t(4;14)+ tumors depend on MMSET expression for their proliferation and that inhibition of MMSET function represents a rational form of therapy targeting against cancers that express high levels of this protein.

## Discussion

### MMSET is a global epigenetic regulator

Deregulation of epigenetic machinery is one of the main drivers of oncogenic transformation and cancer development. While alterations of many epigenetic regulators seem to affect a specific subset of downstream gene targets and pathways, there is a growing number of examples where deregulation of a single component of the machinery affects the global epigenetic landscape, including mutations in *EZH2*, *TET2*, *ASXL1* and *SETD2*, among others [5], [7], [8], [47]–[49]. Besides affecting gene regulation, epigenetic anomalies that change overall chromatin structure might affect other chromatin-dependent processes such as DNA repair and DNA replication.

In t(4;14)+ myeloma, overexpression of MMSET induces a dramatic increase in H3K36 dimethylation throughout the genome. Normally, the H3K36me2 mark is enriched in the 5′ and 3′ proximity of the TSS of highly expressed genes however, the precise role of H3K36me2 in transcriptional regulation is still poorly understood and requires further investigation. Increased methylation levels in the presence of MMSET alter the distribution of this mark, leading to a net decrease in many gene bodies and a significant increase in intergenic regions, with the result being an ∼8-fold overall increase in H3K36me2 levels [27]. Due to dramatic differences in H3K36me2 levels between NTKO and TKO cells, analysis of this type of sequencing data across different conditions presents unique challenges. One possible issue is that ChIP-seq data measures only fractional enrichment and that read numbers do not reflect global levels of H3K36 methylation. We used ChIP-PCR to confirm the patterns of H3K36me2 distribution observed in ChIP-seq analysis (Figures 1E and Supplemental Figures S2A and S2B). These data clearly demonstrate that changes in H3K36me2 distribution observed with ChIP-seq analysis are not artifacts due to a fixed number of reads. Additionally, because of the dramatic changes in H3K36me2 between the NTKO and TKO conditions, it is difficult to find a convincing normalization factor for the reads count. Specifically, when we plotted log of the ratio of the level of H3K27me3 or H3K36me3 in TKO versus NTKO cells, methylation assorted in a normal distribution with most regions showing no change and a equal number of regions showing an increase or decrease in histone methylation (Supplemental Figure S10). By contrast, when the same plot was done for H3K36me2, a bimodal distribution was found with most regions showing a change in H3K36me2 levels with a subset showing a large increase and another set with a large decrease (Supplemental Figure S10). This violates the assumption of most existing normalization methods, which is that the majority of the genome should have similar methylation levels, and makes choosing a normalization factor to use across all loci difficult. For this reason, our analysis is focused mostly on the relative differences of the NTKO/TKO ratio across classes of genes differentially expressed in the two cell lines (Figures 2B and 3A). These distinct patterns (Figures 2B and 3A) would not be affected regardless of the normalization factor used.

Our data also show that the global increase in H3K36 methylation leads to a concomitant genome-wide decrease of H3K27 methylation. This result is in agreement with previous in vitro studies showing that activating histone marks, including H3K36 methylation, antagonize H3K27 methylation through prevention of PRC2 binding to chromatin [35]. Surprisingly, our analysis of H3K27me3 patterns in the presence of high levels of MMSET show that while most of the genome is hypomethylated at this residue due to increased H3K36me2, specific loci, including previously identified Polycomb targets, are hypermethylated on lysine 27 through enhanced recruitment of EZH2. A study by Kalushkova *et al.* showed that Polycomb targets are normally silenced in multiple myeloma cells and our study identifies one possible mechanism explaining how this may be achieved [50].

EZH2/PRC2 complexes are recruited to chromatin via sequence-specific transcription factors [51], through the ability of PRC2 component Jarid2 to bind to DNA [52] and through the ability of the EZH2 accessory protein EED to recognize and bind to the H3K27me3 mark [53]. High levels of MMSET in the t(4;14)+ cells lead to an increased rate of H3K36 methylation, precluding the action of EZH2 and removing potential chromatin binding sites for the PRC2 complex. EZH2 and PRC2 component levels do not change in MMSET-high NTKO cells and thus we propose a model where the PRC2 complex in the nucleus is displaced from many genomic sites (Figure 7E). However, certain loci fail to become hypermethylated on H3K36me2 in MMSET-high cells. Among those, some sites have modest levels of H3K27me3 and EZH2 binding in MMSET-low cells that are further enhanced in MMSET-high cells, while other loci only accumulate appreciable levels of EZH2 and H3K27 in the presence of high levels of MMSET. Many of the EZH2 peaks enhanced and unique to MMSET-high cells sit on CTCF sites, known insulators that block the spread of chromatin marks. We propose that overexpression of MMSET in myeloma plays a role in establishing chromatin boundaries leading to accumulation of EZH2/H3K27me3 and gene repression on one side of the insulator. Previous studies in *Drosophila* showed that genome-wide binding of CTCF aligns with H3K27me3 domains [54] and a very recent study suggests that *Drosophila* MMSET homologue, dMes4, directly interacts with insulator-binding protein Beaf32 and regulates H3K27me3 spreading [55]. Additionally, our model is in agreement with recent work from Gaydos *et al.* showing that in *C. elegans*, H3K36 methylation by MMSET homologue MES-4 antagonizes H3K27 methylation across autosomes and concentrates H3K27me3 on the X chromosome [56]. Loss of *MES-4* expression allows for spreading of the H3K27me3 mark on autosomes and concomitant loss of the mark on the X chromosome. Our findings, as well as those by Gaydos *et al.*, argue that localization of the H3K27 methyl mark greatly depends on the number of genomic loci that are accessible for PRC2 activity. Our identification of CG-rich DNA motifs at sites of enhanced EZH2 enrichment in MMSET-high cells, similar to those previously described to aid in recruitment of EZH2 to chromatin [39], suggests that the underlying DNA sequence also plays a role in specifying genes particularly responsive to EZH2 activity. Furthermore, CpG islands have been shown to recruit KDM2A, an H3K36-specific demethylase, which may provide a suitable, H3K36-demethylated chromatin template for PRC2 binding [57]. Recently, a recurrent mutation in the gene encoding histone H3 isoform *H3.3* was identified in pediatric glioblastoma patients, which converts lysine 27 to methionine [58]. In addition to a genome-wide decrease in H3K27 methylation, as in the case of MMSET overexpression, the H3K27M mutation also induces focal increases in EZH2 and H3K27 methylation and aberrant gene repression. Thus, the mechanism suggested by our study may be applicable to other malignancies characterized by disrupted H3K27 methylation.

### MMSET as a therapeutic target

Multiple studies indicate that high levels of *MMSET* are not exclusive to t(4;14)+ myeloma. Overexpression of *MMSET* also occurs in a number of solid tumors [20], [21] and is correlated with the stage and aggressiveness of the disease. In prostate cancer, the upregulation of *EZH2* in high grade and metastatic disease represses miR-203, which targets *MMSET*, explaining, at least in part, *MMSET* upregulation [59]. Perhaps due to the parallel increase of MMSET and EZH2 in prostate and other tumors, studies to date have not shown a net increase in H3K36 or depression of H3K27me3 in advanced-stage cancers. Nevertheless, we showed that siRNA depletion of *MMSET* in metastatic but not in non-transformed prostatic epithelial cells results in a switch in H3K36/H3K27 methylation, suggesting that metastatic cancer cells may have increased dependency on MMSET for lysine 36 methylation [20]. Additionally, we recently showed that in acute lymphoblastic leukemia, a recurrent mutation within the SET domain of MMSET enhances its methyltransferase activity and induces a global epigenetic change similar to what is observed when MMSET is overexpressed [24].

We and others showed that the histone methyltransferase activity of MMSET is key to its oncogenic potential [11], [12]. However, the full “chromatin switch” driven by MMSET overexpression also depends on the second PWWP domain and the PHD fingers 2, 3 and 4 (Figures 5 and 6). Loss of the second PWWP domain leads to recruitment to chromatin but failure to methylate H3K36. This effect might be due to allosteric interactions between functional domains or improper alignment of the protein on the nucleosome or DNA. In support of this idea, Li *et al.* showed that in vitro methylation activity of MMSET was augmented by the addition of DNA to the reaction mixture or by the use of nucleosomes as a substrate [10]. The loss of the fourth PHD domain is particularly interesting, as this truncation yields an intermediate biological phenotype with an incomplete loss of H3K27 methylation despite a global increase in H3K36me2, albeit to somewhat lower degree than that generated by the WT protein. This region of MMSET was shown to have an affinity for unmethylated histone H3 peptides in vitro, but its deletion, unlike the deletion of the PWWP domain, did not completely block its ability to methylate chromatin [60]. Instead, the resulting partial switch in chromatin was associated with incomplete gene activation and modest growth stimulation, highlighting the importance of H3K27me3/EZH2 dysfunction in the biology of MMSET. The enhanced rate of H3K27me3 demethylation we observed in MMSET-high NTKO cells [27] suggests that MMSET may affect the activity of H3K27me3 demethylases, possibly through the PHD4 domain. Alternatively, the genome-wide distribution or effects of MMSET on H3K36me2 may be qualitatively different with the loss of a domain that helps attract MMSET to chromatin.

*NSD1*, a close homologue of MMSET, is fused to the *NUP98* locus in rare cases of acute myeloid leukemia creating the NUP98-NSD1 fusion protein [61]. Interestingly, the ability of NUP98-NSD1 to transform mouse bone marrow cells and to activate *Hox* gene expression depends on the presence of the analogous fourth PHD finger of the NSD1 moiety [62]. The other PHD domains of MMSET are also critical for its oncogenic function. This was demonstrated by engineering mutations into PHD fingers 2 and 3 analogous to those found in *NSD1* in Sotos syndrome patients [63]. These point mutations of MMSET failed to bind chromatin and failed to alter chromatin methylation. Our findings indicate that PHD domains are additional regions of MMSET that may be considered as therapeutic targets and suggest how these point mutations may inactivate *NSD1* in Sotos syndrome.

The sequencing of the coding regions and genomes of a variety of human tumors showed that mutations in the epigenetic apparatus are among the most common class of alterations in cancer [47], [49], [64], further stimulating interest in epigenetically targeted therapies [1]. While germinal cell lymphoma is associated with gain-of-function mutations of EZH2 [5], multiple myeloma has not been linked directly to alterations in EZH2 function. However, mutations/deletions in the H3K27me3 demethylase *UTX* are observed in multiple myeloma patients [65]. While the role of *UTX* mutations in myelomagenesis is still unclear, it likely involves increases in H3K27 methylation and aberrant gene repression. Thus, the focal increase of H3K27 methylation in the presence of MMSET may have a similar effect as *UTX* mutations, suggesting that EZH2 plays an important, and so far underappreciated, role in multiple myeloma. Prior work implicated EZH2 in myeloma cell proliferation and transformation [66], and our data suggest that t(4;14)+ cells may be particularly sensitive to inhibition of EZH2 (Figure 4F; Supplemental Figure S6A). This may be due to the ability of EZH2i to decrease c-MYC levels in MMSET-overexpressing cells. We previously showed that MMSET increased c-MYC levels by repression of miR126* [43]. Here we show that in MMSET high cells, EZH2 and H3K27me3 accumulate at the miR126 locus. Accordingly, we found that EZH2i stimulates expression of miR126*, which can then directly repress *c*-MYC protein expression (Figures 4G and 4H).

MMSET is commonly misregulated in human cancers and inhibition of MMSET activity may have therapeutic potential for a diverse set of tumors. In tumorigenic prostate cells, MMSET expression maintains the transformed phenotype by stimulating cell growth, migration and invasion [20], [59]. Inhibition of *MMSET* function in MM cells by shRNA in established xenografts led to tumor regression in association with reversal of the chromatin changes. Therefore, we hypothesized that agents that block the enzymatic activity of MMSET or its ability to properly dock with chromatin could represent potential new therapies. Although inhibition of enzymatic activities of proteins such as MMSET and EZH2 is a rational approach, recent success in targeting chromatin-reading domains of BRD4 suggest that inhibition of non-enzymatic domains should also be considered [67]. Indeed, our data suggest that targeting the PHD fingers or PWWP domain may be equally sufficient in preventing MMSET from methylating histones and altering gene expression. Recently identified inhibitors of EZH2 [68], [69] and hopefully soon to be identified inhibitors of MMSET will allow us to determine the therapeutic effects of these targeted therapies on a number of cancer subtypes, including patients with t(4;14) translocations.

## Materials and Methods

### Ethics statement

Animal experiments were approved and in strict compliance with institutional guidelines established by Northwestern University Animal Care and Use Committee (ASP# 2011-1373).

### Tissue culture

All cells were grown in RPMI 1640 (Invitrogen) supplemented with 10% heat-inactivated FBS and 1% penicillin/streptomycin.

### Protein extraction and immunoblotting

Nuclear proteins were extracted using the Nuclear Complex Co-IP Kit (Active Motif). Proteins were electrophoretically separated, blotted and detected using enhanced chemiluminescence. Primary antibodies used were: H3K36me2 (Millipore 07-369), H3K27me3 (Millipore 07-449), MMSET [12], c-MYC (Abcam ab32072), HDAC2 (Millipore 05-814) and pan-H4 (Abcam ab7311). The secondary antibody used was horseradish peroxidase-conjugated donkey anti-rabbit IgG (GE Healthcare Life Sciences).

### Chromatin immunoprecipitation

ChIP experiments for histone modifications and MMSET were performed as described previously [12] using antibodies for H3K36me2 (Millipore, 07-369), H3K36me3 (Abcam, ab9050), H3K27me3 (Millipore, 07-449), MMSET [12], and rabbit IgG (Abcam, ab37415) as a negative control. Histone antibody specificity was confirmed using a MODified Histone Peptide Array (Active Motif), according to the manufacturer's instructions. ChIP-qPCR primers can be found in Supplemental Table S7. *JAM2* promoter primers were previously described [12]. EZH2 ChIP experiments (Cell Signaling, 5246s), were performed with following modification- cells were resuspended in nuclei lysis buffer (10 mM Tris pH 7.5, 10 mM NaCl, 0.2% NP-40, protease inhibitors) for ten minutes, centrifuged, washed and resuspended in SZAK RIPA buffer (150 mM NaCl, 1%v/v Nonidet P-40, 0.5% w/v deoxycholate, 0.1% w/v SDS, 50 mM Tris pH 8, 5 mM EDTA, 0.5 mM PMSF, protease inhibitors) for sonication. Preparation of ChIP libraries and sequencing was performed by the Epigenomics Core at Weill Cornell Medical College. 10 ng of input and ChIP material was processed using the Illumina kit (IP-102-1001). Libraries were loaded onto a HiSeq 2000 or GAIIX at 6 pM, and subjected to 50 or 36 sequencing cycles, respectively. For histone modifications, data from the HiSeq 2000 is presented in the main text and experimental repeats from GAIIAX sequencing are presented in the supplemental figures. Both EZH2 ChIP-seq experiments were sequenced on HiSeq 2000.

### Microarray analysis

Six replicate samples of both NTKO and TKO cells were run on an Illumina microarray (HumanWG-6_V3_0_R0_11282955_A). Among the 48,804 probes on the gene expression array, 28,124 probes were selected for further analysis based on having at least four positive expression values among the six replicates in both NTKO and TKO samples. Based on the Illumina annotation file (http://www.switchtoi.com/annotationfiles.ilmn), a final set of 15,386 protein-coding genes out of the 28,124 selected probes was identified. For each gene, a two-sample t-test was applied to obtain the p-value for significance of differential expression between NTKO and TKO cells. If a gene's expression level (in log scale) was lower than 3 in both NTKO and TKO cells, it was considered as a “not expressed” gene. A two-sample t-test was conducted for each gene not classified as “not expressed”. Genes detected as differentially expressed (p<0.002) were defined up or down modulated according to the sign of t-statistics. All other genes were classified as “not changed” in expression. The GEO accession number for the Illumina gene expression data is GSE57863.

### Histone ChIP-seq data analysis

For histone modifications, the single-end reads were mapped to the human genome (hg19 version) with bowtie 1 (version 0.12.7) by allowing a maximum of 3 mismatches. About 70% to 74% of raw reads were uniquely mapped to the genome for each sample, which were used in the subsequent analysis. Supplemental Table S6 summarizes the count of uniquely mapped reads for each sample. Several reads mapping to the same exact location were considered amplification artifacts and were excluded from the analysis. If there was more than one read mapped to the same genomic location on the same strand, only one read was kept at that location. Supplemental Table S6 shows the updated read number in each sample after removing redundancy. To calculate the read density, each read was extended to the 3′ direction for a distance that approximates the length of the parental DNA fragment from which the short reads derived. To estimate the average length of DNA fragments, we first calculated the cross-correlation between the read frequency on the Watson and Crick strands for each sample. The lag corresponding to the peak point in the cross-correlation function was regarded as the average length of DNA fragments. The read density at any genomic location is defined as the number of extended DNA fragments that cover this given location. To estimate the read count of DNA fragments centered at each position, the 5′ end of each uniquely mapped read was shifted towards the 3′ direction by half fragment size (fragment size as estimated above). To analyze methylation patterns across the genome and compare them between different samples, the read count of each sample was normalized by the average read frequency per base pair of the effective genome size (which was approximately the mappable human genome size and was set as 2.7×10^9^ bp) [70]. After this normalization, the expectation of average read count at any position is 1, and the exact count would show the relative enrichment level compared to average. The average of normalized read counts across all positions in a given genomic window was used as a measure of methylation level within that region. To analyze methylation patterns across the gene body, each gene body was divided evenly into 50 bins regardless of the length of the gene. As such, all genes were perfectly aligned at the TSS and TES. The immediate upstream and downstream 10 k regions were also divided into 50 bins of 200 bp each. The average of normalized read count (defined as above) was used in the comparison of NTKO and TKO samples. To analyze the methylation pattern in the intergenic regions, for each of the 15,386 genes identified from the microarray analysis, if the closest upstream gene was at least 30 kb away from the TSS of the current gene, the intergenic region was selected. This resulted in a total of 6172 intergenic regions. We further ignored the first and last 10 kb of each intergenic region (as these regions are included in the gene plots) and divided the remaining part of each intergenic region evenly into 100 bins. The average of normalized read count per bin was used to compare NTKO samples with TKO samples as described above. The GEO accession number for the histone ChIP-seq data is GSE57977.

### EZH2 ChIP-seq peak detection

All samples were aligned to human genome (hg18) using BWA (version 0.5.8, default parameters). Several reads mapping to the same exact location were considered amplification artifacts and were excluded from the analysis. Each ChIP-seq data set was normalized to its corresponding input lane. ChIP-seq peak calling, genomic annotation of peaks, target genes and comparison of EZH2 peaks in TKO and NTKO cells were performed using ChIPseeqer [71]. The default parameters were used for peak detection (i.e., 2-fold difference between ChIP and INPUT signal, and 10^−15^ statistical significance of the detected peaks). False discovery rates (FDR) for TKO samples were 0.08 and 0.02 for run 1 and run 2, respectively. For NTKO samples, FDR was 0.008 for run 1 and 0.003 for run 2. For both NTKO and TKO samples run 2 peaks were used for downstream analysis. Transcription factor motif analysis was performed using FIRE [72], included in ChIPseeqer, and HOMER [73]. Pathway analysis was performed using iPAGE [74], included in ChIPseeqer. The GEO accession number for the EZH2 ChIP-seq data is GSE57632.

### Read density tracks

Single-end reads were aligned with bowtie 1 by allowing a maximum of 3 mismatches. The read density tracks were made using the HOMER tool (version 3.11 using default parameters), where each sample was normalized into 10 million reads [73].

### RNA-seq

Total RNA from KMS11 or TKO cells treated with 2 µM of GSK343 or GSK669 for seven days was sequenced on HiSeq 2000. RNA-seq samples were aligned to hg19 using the STAR aligner (v2.3.0). For the calculation of FPKM values, cuffdiff was used (v2.1.2) and the UCSC hg19 annotation from igenomes (the latest annotation to use: http://cufflinks.cbcb.umd.edu/igenomes.html). Pathway analysis was performed using iPAGE [74]. The GEO accession number for the RNA-seq data is GSE57478.

### Gene Set Enrichment Analysis (GSEA)

GSEA 2.0 with default parameters was used to identify the enrichment of previously defined signatures among genes upregulated in TKO cells.

### RNA extraction, cDNA synthesis, and qRT-PCR

RNA was extracted from cells using the RNeasy Plus Mini Kit (Qiagen). cDNA was synthesized from total RNA using the iScript cDNA Synthesis Kit (Bio-Rad). Quantitative RT-PCR was performed using predesigned TaqMan assays (Applied Biosystems) for *JAM2* (Hs00221894_m1), *JAM3* (Hs00230289_m1), *DLL4* (Hs00184092_m1), *CA2* (Hs00163869_m1), *CR2* (Hs00153398_m1), *CDCA7* (Hs00230589_m1), *LTB* (Hs00242739_m1) and normalized to a *GAPDH* (Hs99999905_m1) control. qRT-PCR was run on a LightCycler 480II (Roche).

### EZH2 inhibitor treatment

1×10^5^ cells were plated in the presence of 1 µM, 2 µM or 4 µM of GSK343 or GSK669 as a control. After seven days, cells were counted and proteins extracted for immunoblotting.

### miRNA expression analysis

To determine miRNA expression levels, total RNA was isolated using the miRNeasy Mini Kit (Qiagen). The miRCURY LNA Universal RT microRNA PCR kit (Exiqon) was used for reverse transcription and miRNA amplification. Expression was calculated using the ^ΔΔ^CT method and U6 was used as internal controls. Primers for miR-126* (cat. 204584) and U6 (cat. 203907) were purchased from Exiqon.

### Cloning and site-directed mutagenesis

All of the MMSET constructs were cloned into the pRetroX-DsRed vector (Clontech) except PHD1-M2 and PHD2-M2, which were cloned into pRetroX-ZsGreen (Clontech). A nuclear localization signal was inserted at the N-terminus of PHD1-M2 and PHD2-M2 constructs. Site-directed mutagenesis of PHD fingers 2 and 3 was performed using QuickChange Lighting Site-Directed Mutagenesis Kit (Stratagene) following the manufacturer's recommendations.

### Retroviral infection

For the repletion system, TKO cells were transduced with retroviral vectors harboring MMSET or mutant isoforms. All retroviruses were produced by transfection of amphotropic 293T cells with appropriate plasmids and FuGENE 6 Transfection reagent (Roche). After infection, cells were sorted by flow cytometry using the DsRed protein marker and expanded in culture for further studies.

### Colony formation assay

2×10^3^ cells were grown in 1 mL of semisolid methylcellulose medium (MethoCult H4100; StemCell Technologies Inc.) supplemented with complete medium and heat-inactivated FBS. Two weeks later, colonies were counted in at least 6 random fields.

### Cell growth

3×10^4^ cells were grown in a 6-well plate with 2 mL of complete medium. Live cells were collected and counted at indicated days using trypan blue dye.

### Mouse xenograft model

Six-week-old female C57BL6 Nu/Nu mice were obtained from The Jackson Laboratory and were acclimated for at least for 24 h before tumor cell injection. A total of 5×10^6^ KMS11 cells harboring an inducible *MMSET* shRNA were resuspended in 100 µL cold PBS and were mixed with 100 µL of CultreX PathClear BME (3432-005-02, Trevigen). The mixture was injected subcutaneously in the dorsal region next to both thighs. One week after injection, mice were divided in two groups (n = 5 per group). The control group was administered regular water and the treatment group was given doxycycline 2 mg/mL in water containing 0.04 g/mL of sucrose. The water was changed every other day to ensure delivery of a stable dose of doxycycline. Two weeks after treatment initiation, images were acquired using IVIS^R^ Spectrum (Caliper Life Sciences, Inc.). For imaging, firefly Luciferin (150 mg/kg) (Gold Biotechnology) was injected intraperitoneally and images were taken 10–15 min later. Bioluminiscence was quantified using Living Images software (Caliper Life Sciences, Inc.). GraphPad Prism software was used for survival analysis.

For protein extraction, tumor samples were immediately frozen in liquid nitrogen and stored at −80°C. Frozen tumors were mechanically homogenized using a biopulverizer (Biospec) chilled at −80°C, and incubated in lysis buffer (10 mM Hepes ph 7.9, 10 mM KCl, 1.5 mM MgCl_2_, 0.5% NP40, 1 mM PMSF, 1 mM DTT, proteinase inhibitors) on ice for 20 min. Upon centrifugation, the supernatant containing the cytoplasmic fraction was discarded and nuclei were resuspended in lysis buffer containing 20 mM Hepes pH 7.9, 400 mM NaCl, 1.5 mM MgCl_2_, 0.2 mM EDTA, 15% glycerol, 1 mM PMSF, 1 mM DTT and proteinase inhibitors. Lysates were incubated at 4°C for 20 min on an orbital rotator and further sonicated for 20 min using a Bioruptor (Diagenode, Inc) (30 seconds on, 30 seconds off). The supernatant containing nuclear proteins was analyzed by immunoblot.

## Supporting Information

Figure S1 (A) Heatmaps of H3K36me2 distribution between NTKO (left) and TKO (right) cells using replicate samples. Data were plotted as in Figure 1B. (B) Average read density across 15,386 genes and 6,172 intergenic regions using ChIP-seq replicate samples.
(TIF)

Figure S2 (A) ChIP-qPCR for H3K36me2 on *GAPDH* locus. Methylation enrichment was tested on the promoter (TSS) and on the regions upstream (5′) and downstream (3′) from the TSS. Two independent biological replicates are shown. (B) ChIP-qPCR on *SNX16* locus. H3K36me2 (top) and H3K27me3 (bottom) enrichment was tested on the promoter (TSS) and on the regions upstream (5′) and downstream (3′) from the TSS. Two independent biological replicates are shown.
(TIF)

Figure S3 (A) ChIP-qPCR on *CR2* locus. H3K36me2 (top) and H3K27me3 (bottom) enrichment was tested on the promoter (TSS) and on the regions upstream (5′) and downstream (3′) from the TSS. Two independent biological replicates are shown. (B) Heatmaps of H3K36me3 distribution in ChIP-seq replicate samples in NTKO (left) and TKO (right) cells. Data were plotted as in Figure 1B.
(TIF)

Figure S4 (A) ChIP-qPCR for H3K36me2 on *CDCA7* gene. Methylation enrichment was tested on the promoter (TSS) and on the regions upstream (5′) and downstream (3′) from the TSS. Two independent biological replicates are shown. (B) ChIP-qPCR for H3K36me2 (left) and H3K27me3 (right) on the promoter of *DLL4* gene. Two independent biological replicates are shown.
(TIF)

Figure S5 (A) Correlation plot of two independent EZH2 ChIP-seq experiments in NTKO (left) and TKO (right) cells. (B) Box plot representing average height (left) or average length (right) of EZH2 peaks common in both NTKO and TKO cells. Statistical significance was determined using Welch Two Sample t-test (*** p<1e-6). (C) Motif analysis using FIRE [72] identified sequences enriched in EZH2-bound peaks in NTKO cells. (D) Immunoblot of nuclear extracts from KMS11 and TKO cells treated with 1 µM or 2 µM GSK343 for seven days. Inactive compound GSK669 was used as a control.
(TIF)

Figure S6 (A) Relative cell number of MMSET-high (KMS28, OPM2) and MMSET-low (Karpas 620, ARP1) myeloma cells treated with indicated doses of the EZH2 small molecule inhibitor (GSK343) for seven days. Inactive compound, GSK669, was used as a control. The graph represents three independent experiments +/− standard deviation. (B) UCSC genome browser of H3K27me3 and EZH2 enrichment on miR-126* locus (C) Heat map of over-represented gene categories among genes differentially expressed (≥2-fold) in MMSET-high (KMS11) or MMSET-low (TKO) cells upon seven days of GSK343 treatment. Enrichment was measured using iPAGE analysis [74].
(TIF)

Figure S7 (A) Quantitative RT-PCR for *CR2* using RNA from the TKO repletion experiment. Experiment was performed in duplicate and graph represents average gene expression +/− standard deviation. (B) Growth curve of TKO cells repleted with either empty vector, wild-type MMSET or MMSET lacking PHD4. Experiment was performed in triplicate and graph represents average cell growth +/− standard deviation. (C) Images of the colony forming assay using repleted TKO cells. (D) Mass spectrometry analysis of H3K27 and H3K36 methylation from TKO cells expressing vector control, wild-type MMSET or enzymatically inactive Y1118A mutant MMSET. Analysis was performed as previously described [27]. (E) Growth curve of TKO cells infected with vector control, wild-type MMSET or MMSET lacking the second PWWP domain. Experiment was performed in triplicate and graph represents average cell growth +/− standard deviation.
(TIF)

Figure S8 (A) Colony forming assay, using TKO cells repleted with vector control, wild-type MMSET, PHD1-M2 or PHD2-M2 constructs. Experiment was performed in triplicate and at least six different fields were counted. Graph represents average colony count +/− standard deviation. (B) Image of the colony assay using TKO cells repleted with vector control, wild-type MMSET or MMSET mutated at cysteines 720 or 857.
(TIF)

Figure S9 Xenograft mice after four weeks of treatment. Three mice on the left were untreated while the three mice on the right received doxycycline in their drinking water.
(TIF)

Figure S10 Log2ratio plot of H3K27me3, H3K36me3 and H3K36me2 methylation in NTKO and TKO cells. Normalization factors were calculated using EdgeR with default parameters.
(TIF)

Table S1 Differentially expressed genes in NTKO and TKO cells.
(TXT)

Table S2 List of genes bound by EZH2 in NTKO cells only, TKO cells only or bound in both cell types.
(TXT)

Table S3 Over-represented gene categories among genes bound by EZH2 in NTKO and TKO cells.
(TXT)

Table S4 List of differentially expressed genes (≥2-fold) upon EZH2 inhibition in KMS11 and TKO cells.
(TXT)

Table S5 Over-represented gene categories among differentially expressed genes (≥2-fold) upon EZH2 inhibition in KMS11 and TKO cells.
(TXT)

Table S6 Numbers of ChIP-seq reads.
(DOCX)

Table S7 Primer sequences.
(TXT)
