# Supplementary figures and images for: Histone Methyltransferase MMSET/NSD2 Alters EZH2 Binding and Reprograms the Myeloma Epigenome through Global and Focal Changes in H3K36 and H3K27 Methylation
Source: PLoS Genet. 2014 Sep 4;10(9):e1004566. doi: 10.1371/journal.pgen.1004566 (PMC4154646; doi:10.1371/journal.pgen.1004566)

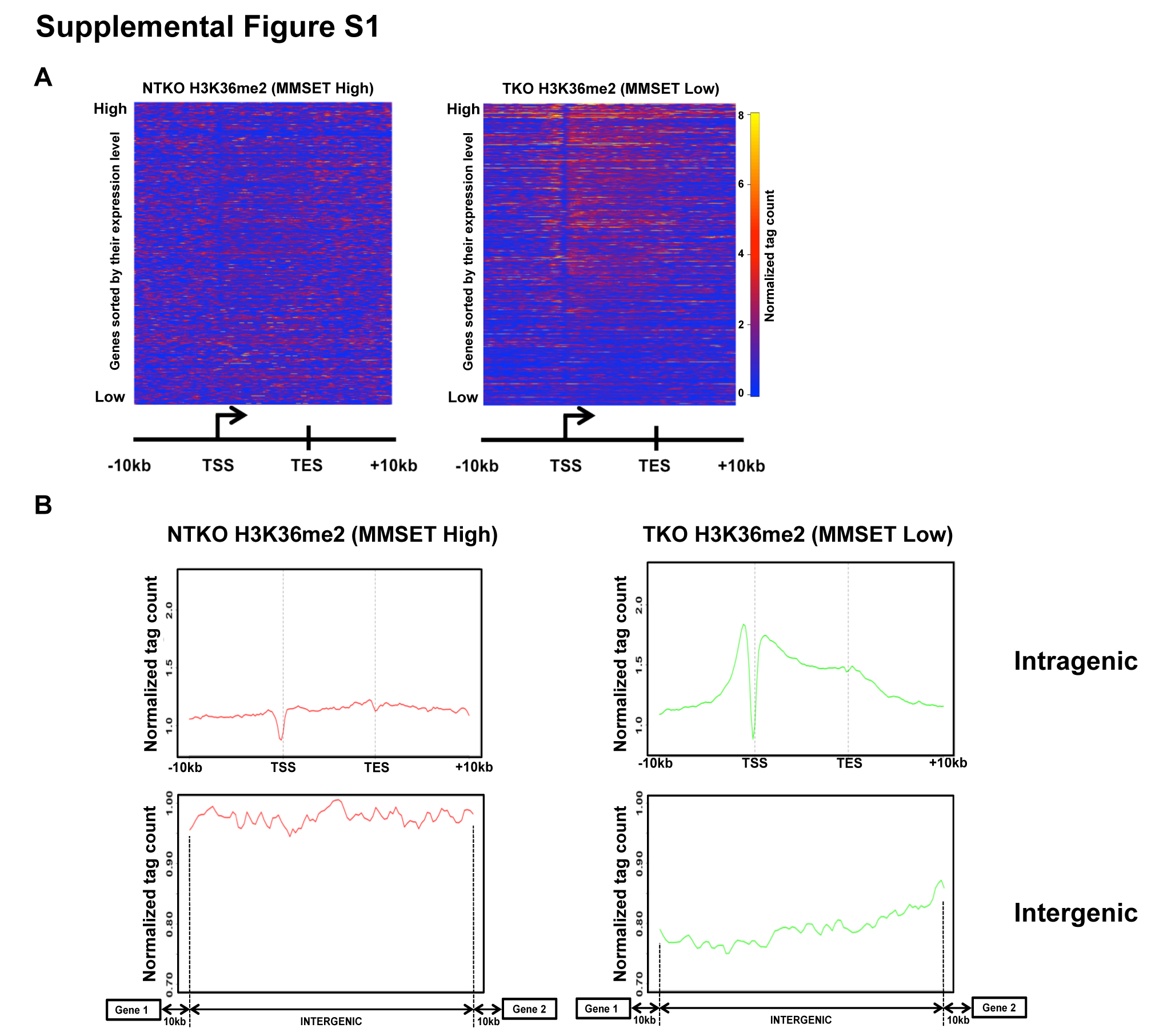

Supplement: Figure S1 — (A) Heatmaps of H3K36me2 distribution between NTKO (left) and TKO (right) cells using replicate samples. Data were plotted as in Figure 1B. (B) Average read density across 15,386 genes and 6,172 intergenic regions using ChIP-seq replicate samples. (TIF) [file pgen.1004566.s001.tif]

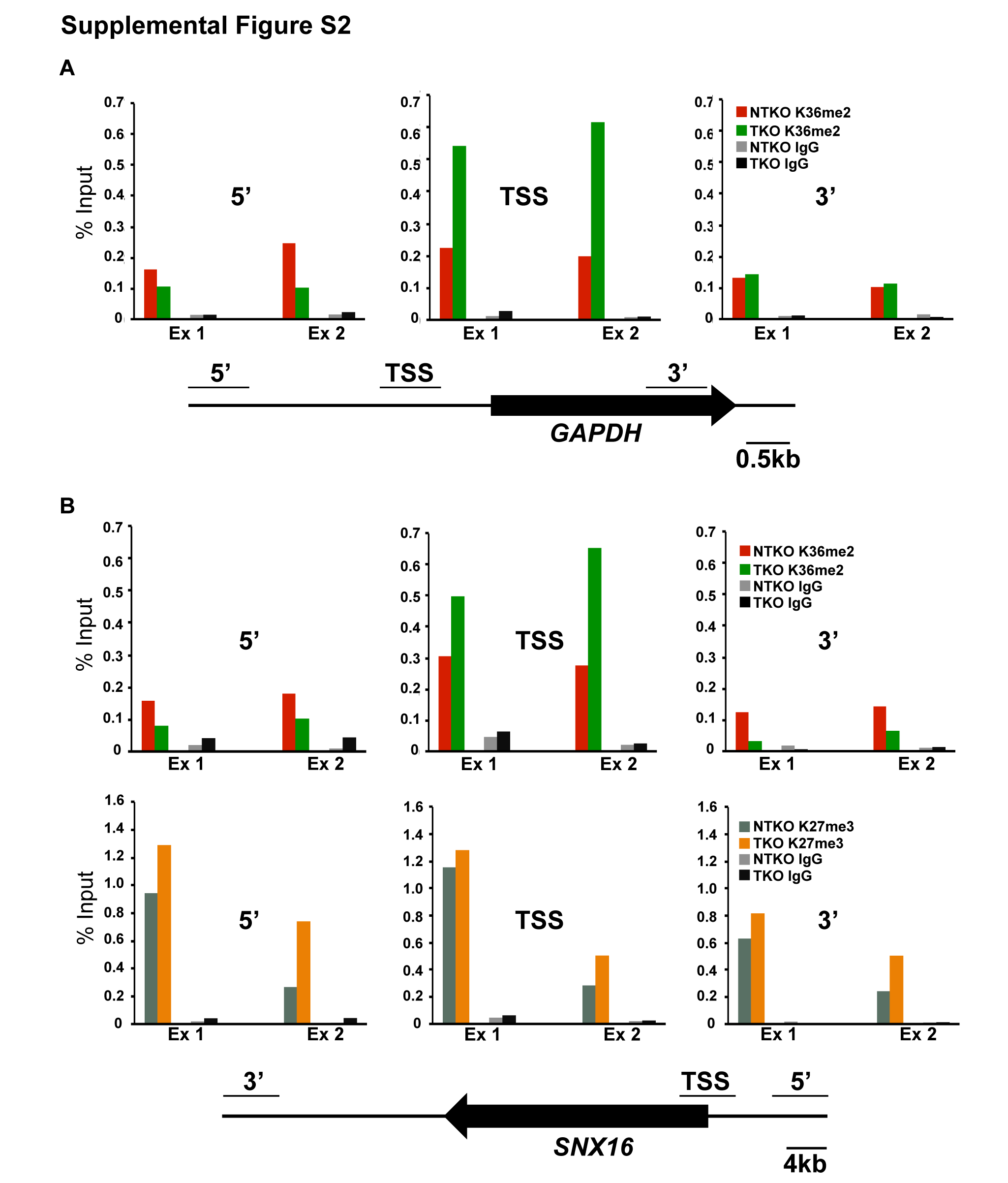

Supplement: Figure S2 — (A) ChIP-qPCR for H3K36me2 on GAPDH locus. Methylation enrichment was tested on the promoter (TSS) and on the regions upstream (5′) and downstream (3′) from the TSS. Two independent biological replicates are shown. (B) ChIP-qPCR on SNX16 locus. H3K36me2 (top) and H3K27me3 (bottom) enrichment was tested on the promoter (TSS) and on the regions upstream (5′) and downstream (3′) from the TSS. Two independent biological replicates are shown. (TIF) [file pgen.1004566.s002.tif]

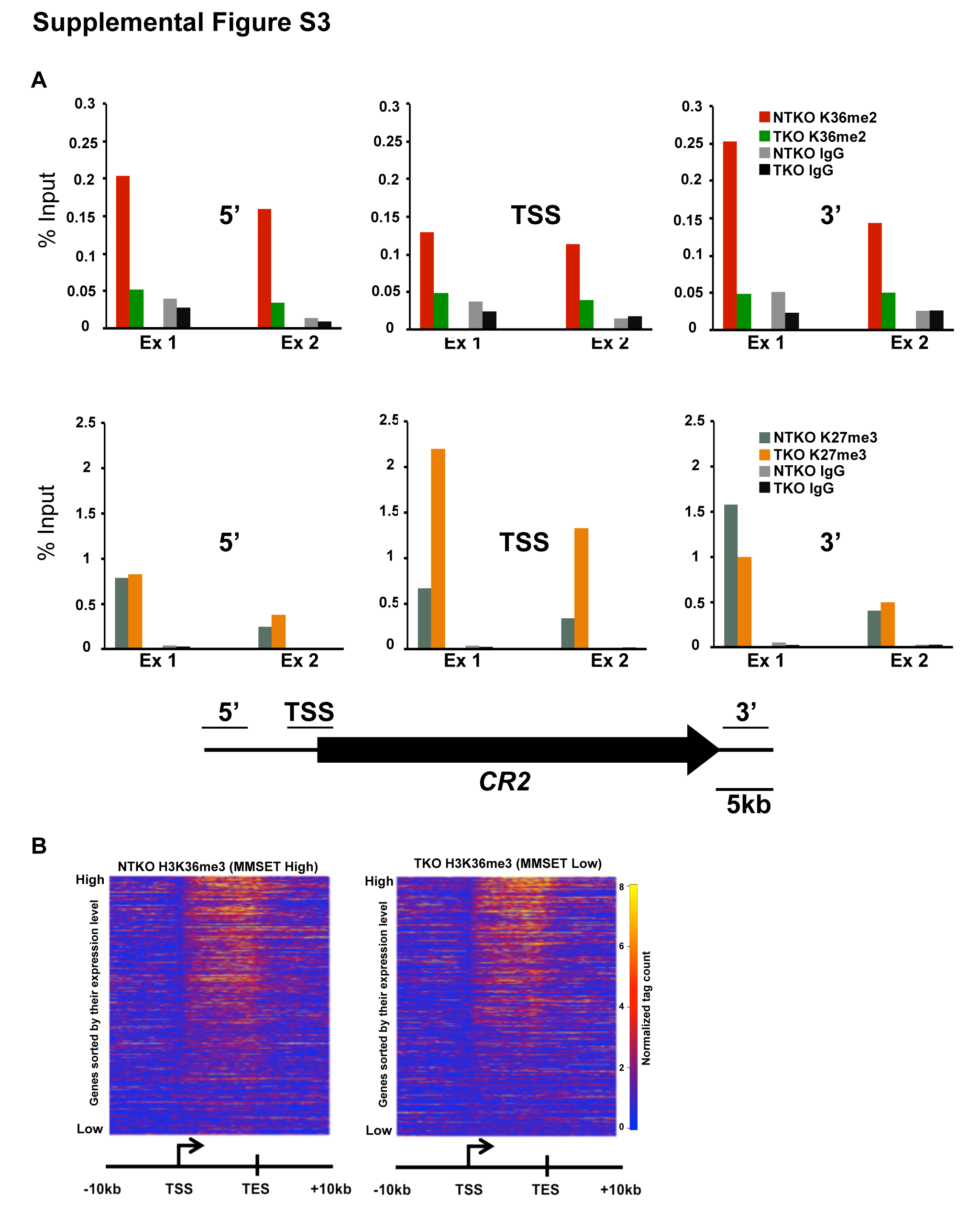

Supplement: Figure S3 — (A) ChIP-qPCR on CR2 locus. H3K36me2 (top) and H3K27me3 (bottom) enrichment was tested on the promoter (TSS) and on the regions upstream (5′) and downstream (3′) from the TSS. Two independent biological replicates are shown. (B) Heatmaps of H3K36me3 distribution in ChIP-seq replicate samples in NTKO (left) and TKO (right) cells. Data were plotted as in Figure 1B. (TIF) [file pgen.1004566.s003.tif]

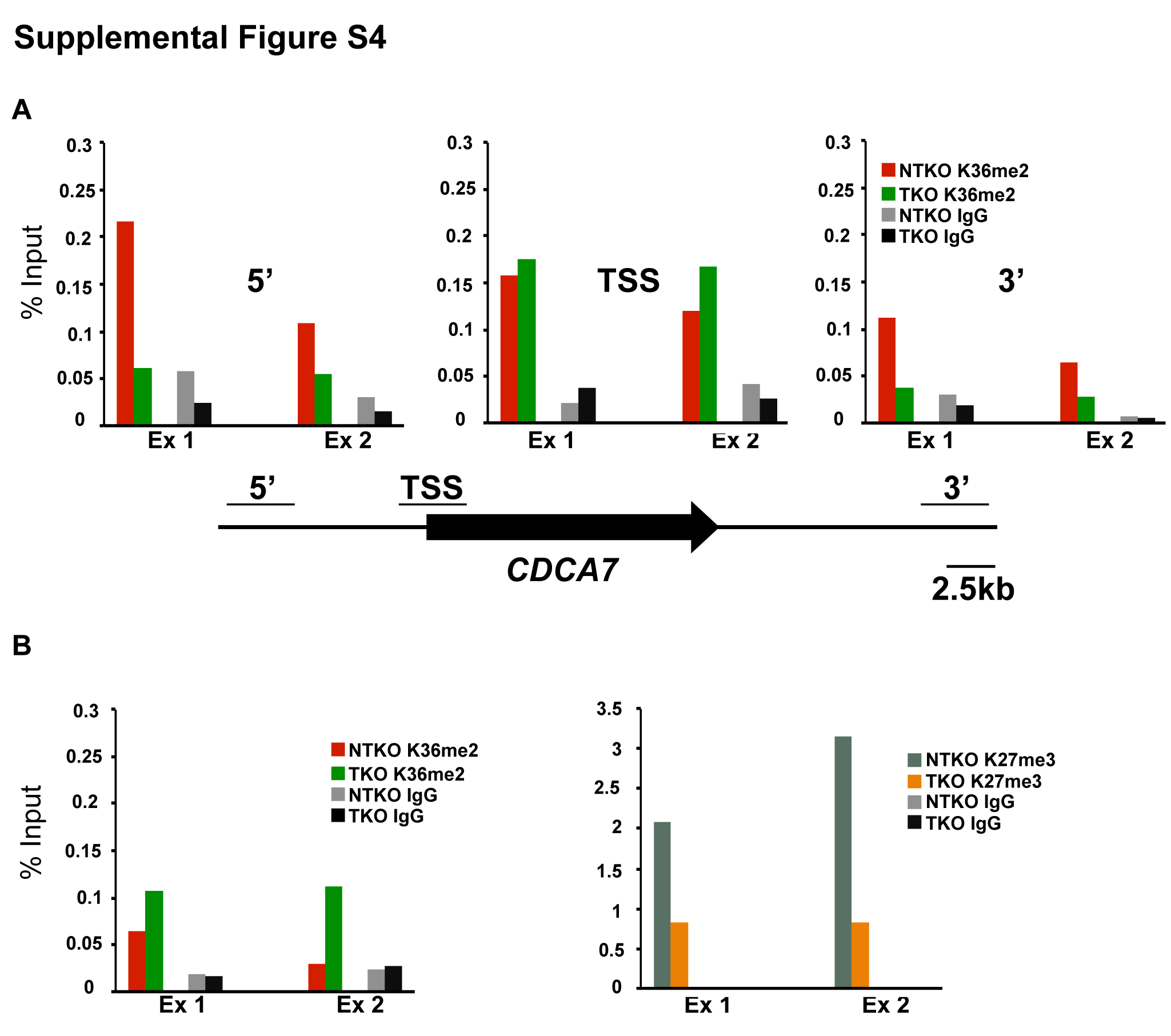

Supplement: Figure S4 — (A) ChIP-qPCR for H3K36me2 on CDCA7 gene. Methylation enrichment was tested on the promoter (TSS) and on the regions upstream (5′) and downstream (3′) from the TSS. Two independent biological replicates are shown. (B) ChIP-qPCR for H3K36me2 (left) and H3K27me3 (right) on the promoter of DLL4 gene. Two independent biological replicates are shown. (TIF) [file pgen.1004566.s004.tif]

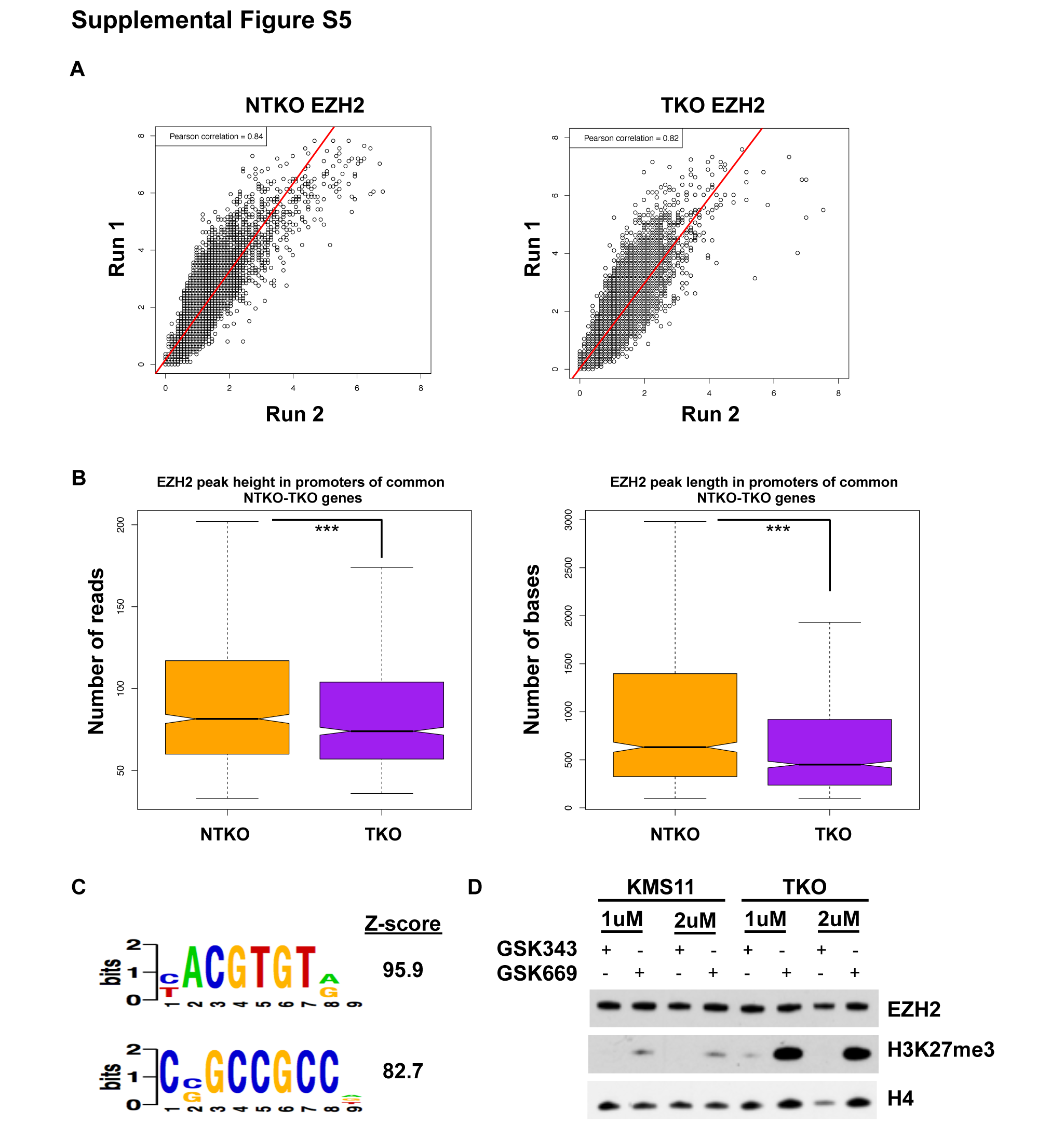

Supplement: Figure S5 — (A) Correlation plot of two independent EZH2 ChIP-seq experiments in NTKO (left) and TKO (right) cells. (B) Box plot representing average height (left) or average length (right) of EZH2 peaks common in both NTKO and TKO cells. Statistical significance was determined using Welch Two Sample t-test (*** p<1e-6). (C) Motif analysis using FIRE [72] identified sequences enriched in EZH2-bound peaks in NTKO cells. (D) Immunoblot of nuclear extracts from KMS11 and TKO cells treated with 1 µM or 2 µM GSK343 for seven days. Inactive compound GSK669 was used as a control. (TIF) [file pgen.1004566.s005.tif]

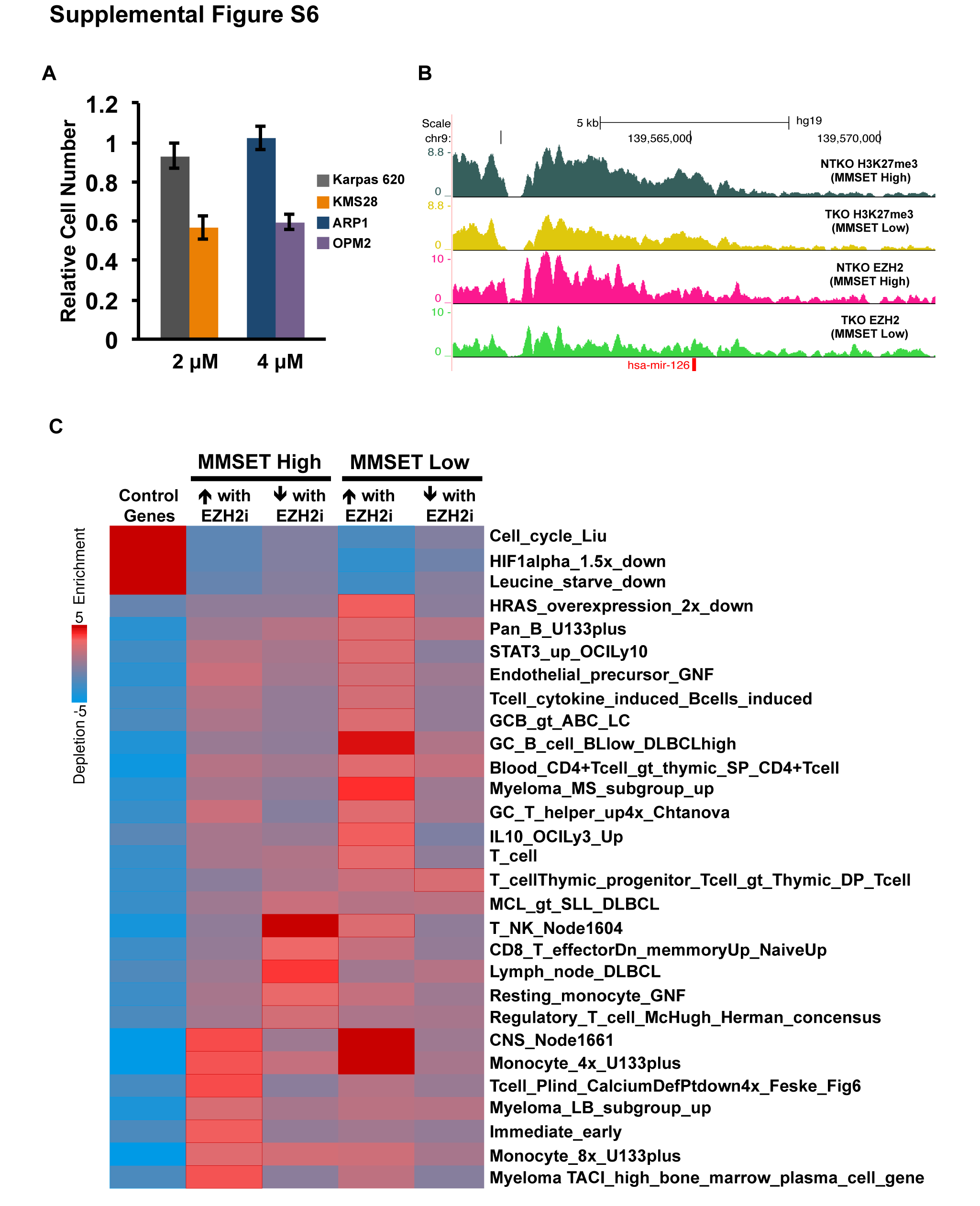

Supplement: Figure S6 — (A) Relative cell number of MMSET-high (KMS28, OPM2) and MMSET-low (Karpas 620, ARP1) myeloma cells treated with indicated doses of the EZH2 small molecule inhibitor (GSK343) for seven days. Inactive compound, GSK669, was used as a control. The graph represents three independent experiments +/− standard deviation. (B) UCSC genome browser of H3K27me3 and EZH2 enrichment on miR-126* locus (C) Heat map of over-represented gene categories among genes differentially expressed (≥2-fold) in MMSET-high (KMS11) or MMSET-low (TKO) cells upon seven days of GSK343 treatment. Enrichment was measured using iPAGE analysis [74]. (TIF) [file pgen.1004566.s006.tif]

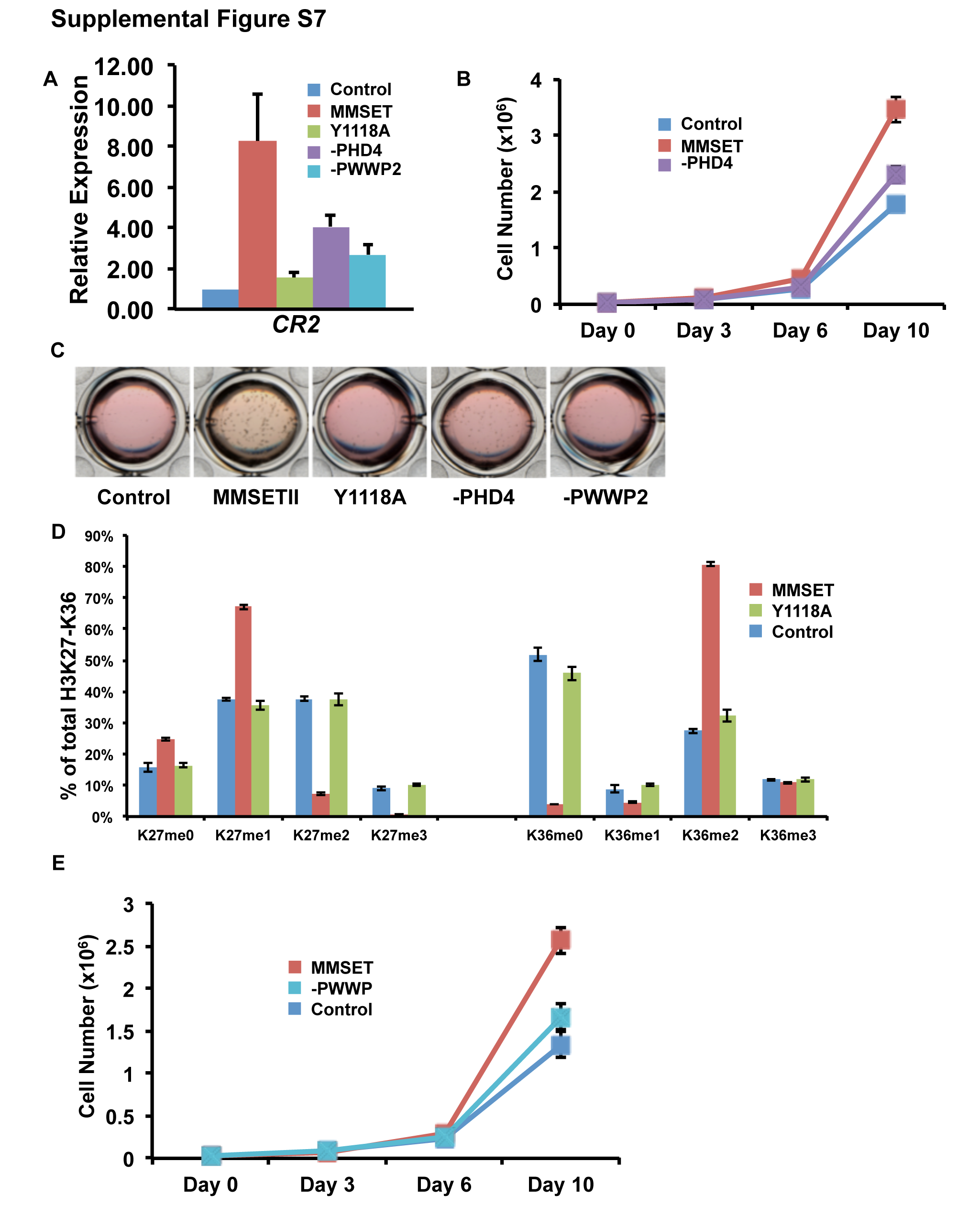

Supplement: Figure S7 — (A) Quantitative RT-PCR for CR2 using RNA from the TKO repletion experiment. Experiment was performed in duplicate and graph represents average gene expression +/− standard deviation. (B) Growth curve of TKO cells repleted with either empty vector, wild-type MMSET or MMSET lacking PHD4. Experiment was performed in triplicate and graph represents average cell growth +/− standard deviation. (C) Images of the colony forming assay using repleted TKO cells. (D) Mass spectrometry analysis of H3K27 and H3K36 methylation from TKO cells expressing vector control, wild-type MMSET or enzymatically inactive Y1118A mutant MMSET. Analysis was performed as previously described [27]. (E) Growth curve of TKO cells infected with vector control, wild-type MMSET or MMSET lacking the second PWWP domain. Experiment was performed in triplicate and graph represents average cell growth +/− standard deviation. (TIF) [file pgen.1004566.s007.tif]

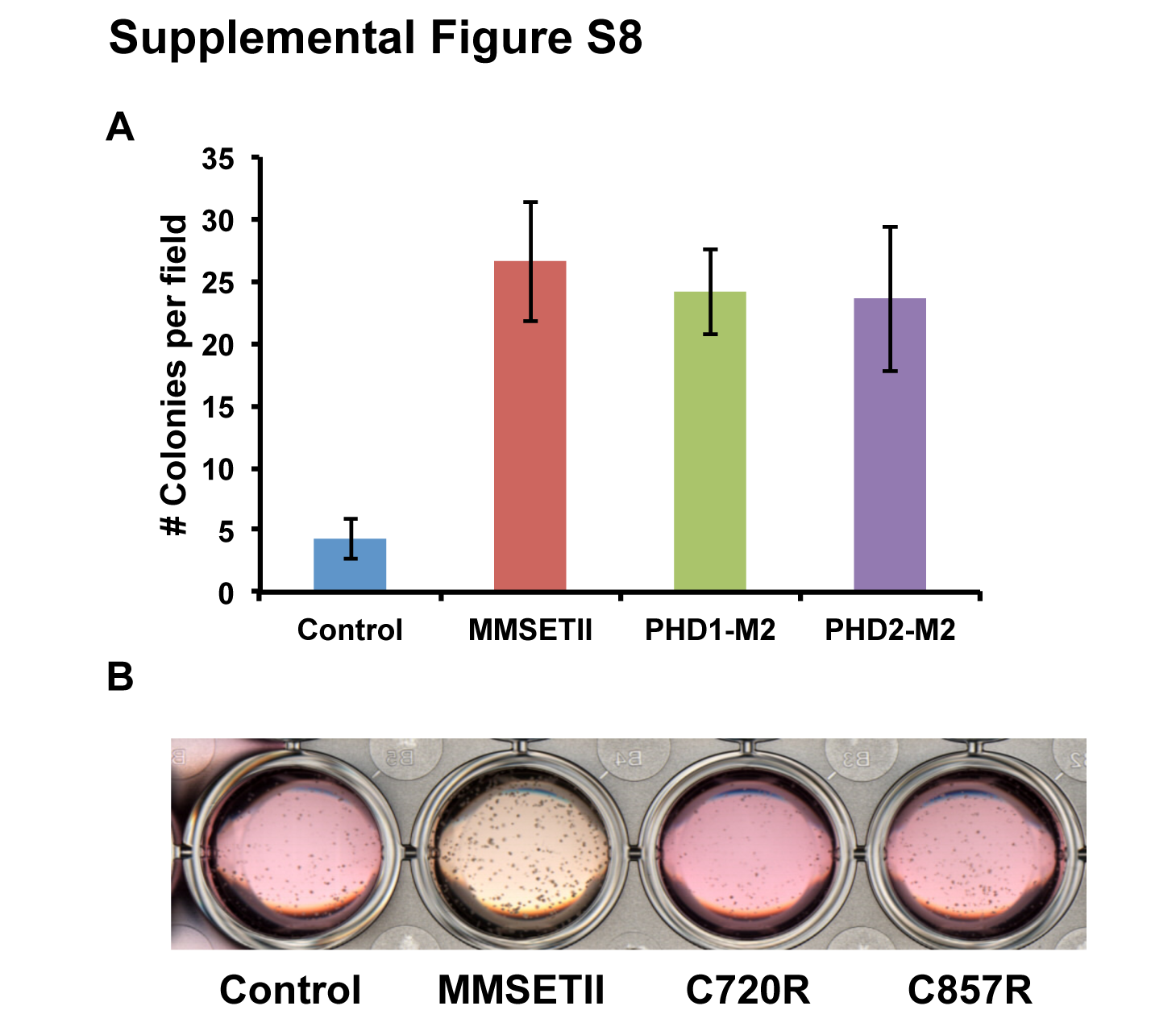

Supplement: Figure S8 — (A) Colony forming assay, using TKO cells repleted with vector control, wild-type MMSET, PHD1-M2 or PHD2-M2 constructs. Experiment was performed in triplicate and at least six different fields were counted. Graph represents average colony count +/− standard deviation. (B) Image of the colony assay using TKO cells repleted with vector control, wild-type MMSET or MMSET mutated at cysteines 720 or 857. (TIF) [file pgen.1004566.s008.tif]

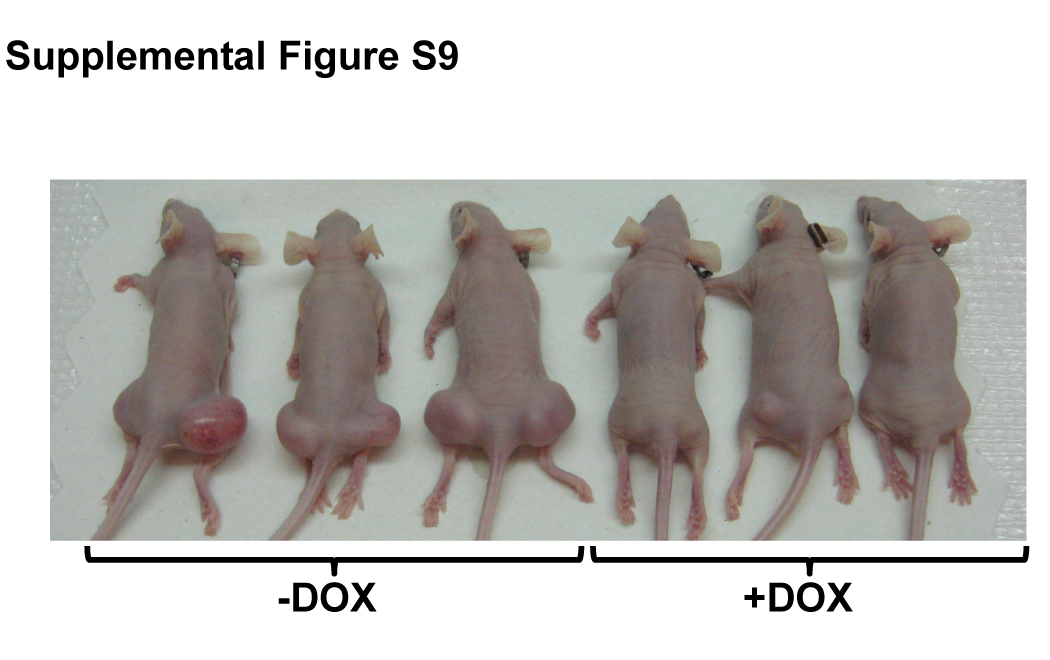

Supplement: Figure S9 — Xenograft mice after four weeks of treatment. Three mice on the left were untreated while the three mice on the right received doxycycline in their drinking water. (TIF) [file pgen.1004566.s009.tif]

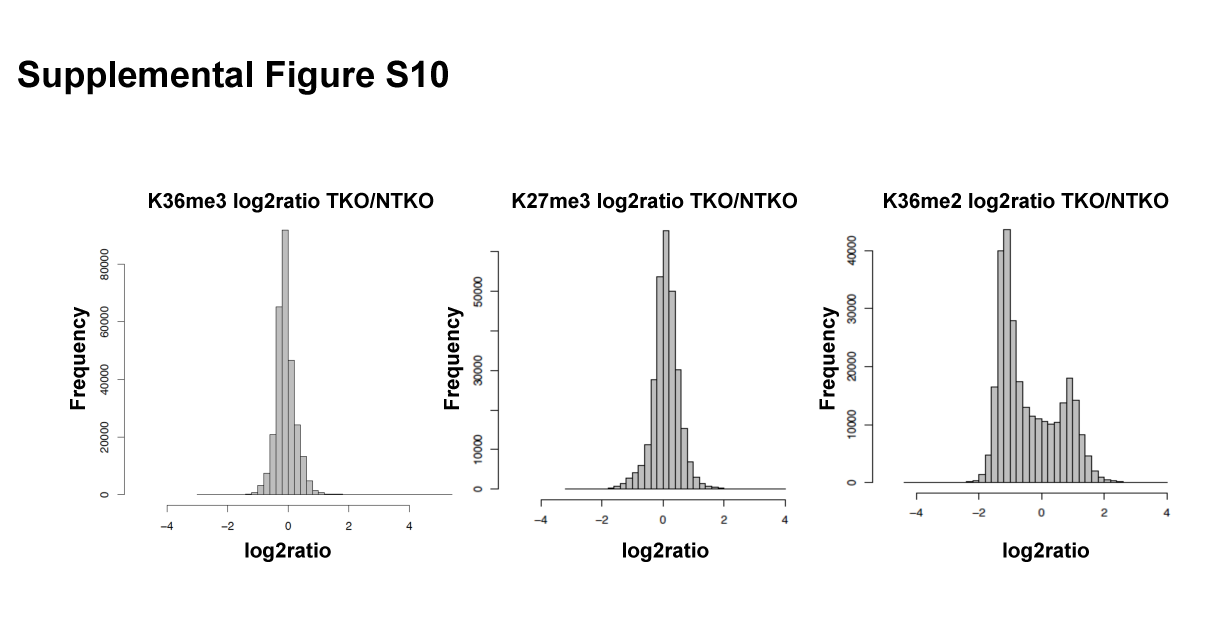

Supplement: Figure S10 — Log2ratio plot of H3K27me3, H3K36me3 and H3K36me2 methylation in NTKO and TKO cells. Normalization factors were calculated using EdgeR with default parameters. (TIF) [file pgen.1004566.s010.tif]
