# Supplementary material for: Histone Methyltransferase MMSET/NSD2 Alters EZH2 Binding and Reprograms the Myeloma Epigenome through Global and Focal Changes in H3K36 and H3K27 Methylation
Source: PLoS Genet. 2014 Sep 4;10(9):e1004566. doi: 10.1371/journal.pgen.1004566 (PMC4154646; doi:10.1371/journal.pgen.1004566)
Supplement: Table S6 — Numbers of ChIP-seq reads. (DOCX) [file pgen.1004566.s016.docx]

**Table S6. ChIP-seq read numbers (in million)**
